# Inhibition of *Pseudomonas aeruginosa* quorum sensing by methyl gallate from *Mangifera indica*

**DOI:** 10.1038/s41598-023-44063-0

**Published:** 2023-10-20

**Authors:** Nourhan G. Naga, Ahmed A. Zaki, Dalia E. El-Badan, Heba S. Rateb, Khaled M. Ghanem, Mona I. Shaaban

**Affiliations:** 1 Botany and Microbiology Department, Faculty of Science, Alexandria University, Alexandria, Egypt; 2 Pharmacognosy Department, Faculty of Pharmacy, Mansoura University, El Mansoura, Egypt; 3 Department of Biological Sciences, Faculty of Science, Beirut Arab University, Beirut, Lebanon; 4 Department of Pharmaceutical and Medicinal Chemistry, Faculty of Pharmacy, Misr University for Science and Technology, Cairo, Egypt; 5 Microbiology and Immunology Department, Faculty of Pharmacy, Mansoura University, El Mansoura, Egypt

**Keywords:** Biotechnology, Microbiology, Plant sciences

## Abstract

Antipathogenic drugs are a potential source of therapeutics, particularly following the emergence of multiple drug-resistant pathogenic microorganisms in the last decade. The inhibition of quorum sensing (QS) is an advanced antipathogenic approach for suppression of bacterial virulence and dissemination. This study aimed to investigate the inhibitory effect of some Egyptian medicinal plants on the QS signaling system of *Pseudomonas aeruginosa*. Among the tested plants, *Mangifera indica* exhibited the highest quorum sensing inhibition (QSI) activity against *Chromobacterium violaceum* ATCC 12472. Four pure compounds were extracted and identified; of these, methyl gallate (MG) showed the most potent QSI. MG had a minimum inhibitory concentration (MIC) of 512 g/mL against *P. aeruginosa* strains PAO1, PA14, Pa21, Pa22, Pa23, Pa24, and PAO-JP2. The virulence factors of PAO1, PA14, Pa21, Pa22, Pa23, and Pa24 were significantly inhibited by MG at 1/4 and 1/2 sub-MICs without affecting bacterial viability. Computational insights were performed by docking the MG compound on the LasR receptor, and the QSI behavior of MG was found to be mediated by three hydrogen bonds: Trp60, Arg61, and Thr75. This study indicates the importance of *M. indica* and MG in the inhibition and modulation of QS and QS-related virulence factors in *P. aeruginosa*.

## Introduction

*Pseudomonas aeruginosa* is an opportunistic human pathogenic bacterium that is Gram-negative, encapsulated, rod-shaped, and can be isolated from a variety of ecosystems^1^. It is capable of colonizing critical body organs, causing life-threating infections^2^ especially in immune-compromised patients such as AIDS, cystic fibrosis, cancer patients and severe burn victims^3^. It can build a biofilm that serves as a protective barrier, blocking antimicrobials from reaching the pathogen and leading to multiple drug resistance (MDR) to a wide range of antimicrobials. Therefore, the development of innovative solutions to this problem is urgently required^4,5^.

In addition to biofilm formation, *P. aeruginosa* possesses various virulence factors such as alginate, lipase, protease, chitinase, aminopeptidase, and elastase, which aid in microbial dissemination. Quorum sensing (QS) regulates this virulence behavior by the release of signaling molecules known as autoinducers^6,7^. Autoinducers support cell–cell communication and cross-talk between two different species that coexist in the same area^8^. In *P. aeruginosa,* four fundamental QS pathways have been identified. The first is known as the LasI/LasR cascade. The second includes the RhlI/RhlR pathway^9^. The third is the *Pseudomonas* quinolone signal (PQS)^10^. The fourth is the AI-based integrated quorum sensing system^11^. These four signaling systems form a network that is connected and hierarchically regulates itself as the systems reach the threshold level and control the expression of QS-regulated virulence factors^12^.

One of the most promising strategies for reducing *Pseudomonas* pathogenicity is quorum sensing inhibition (QSI)^5,13^. Many natural QSIs have been reported; for example, luteolin compound^14^, coumarin^15^, ginseng^16^, clove oil^17^, butein, and sappanol^18^. Synthetic QSIs such as meloxicam and piroxicam^19^, diarylheptanoids^20^, benzamide-benzimidazole^21^, and aspirin^22^ have also been studied.

Plants have long been considered the most potent source of therapeutics, and have drawn attention for their QSI potential. The plant ecosystem contains high bacterial density, so plants have diverse defensive mechanisms against infection^23^. The aim of this study was to assess the QSI potential of several medicinal plants and purify the products responsible for this activity. The effect of these pure compounds on QSI was evaluated against various *Pseudomonas* virulence factors. Additionally, molecular docking analysis was performed to verify the potential activity of MG on the LasR receptor.

## Results

### QSI activity of crude extracts

*Mangifera indica* crude extract exhibited the highest QSI activity, followed by *P. tortuous*, *A. halimus*, *E. paralias*, and *E. azurea,* with a diameter of 24, 19, 17, 16, and 14 mm, respectively. In contrast, *R. raetam* did not show any QSI after incubation for 48 h at 28 °C using an LB medium double-layer culture plate.

### Structure clarification and fractionation of isolated compounds from *M. indica*

The chromatographic analysis of the *M. indica* ethyl acetate extract led to the identification of four known phenolic components. By examining NMR and HRMS data and comparing it to spectroscopic data previously published in the literature, compound structural identities were confirmed.

The compounds (Fig. 1) were identified as iriflophenone mono-O-galloyl-glucoside (**1**) (Figs. S1–S7)^24–26^, iriflophenone 3-C-(6-O-p-hydroxybenzoyl)-β-d-glucoside (**2**) (Figs. S8–S14)^26,27^, MG (**3**) (Figs. S15–S18)^24^, and penta-O-galloyl-glucoside (**4**) (Figs. S19–S22)^24^.

**Figure 1:**
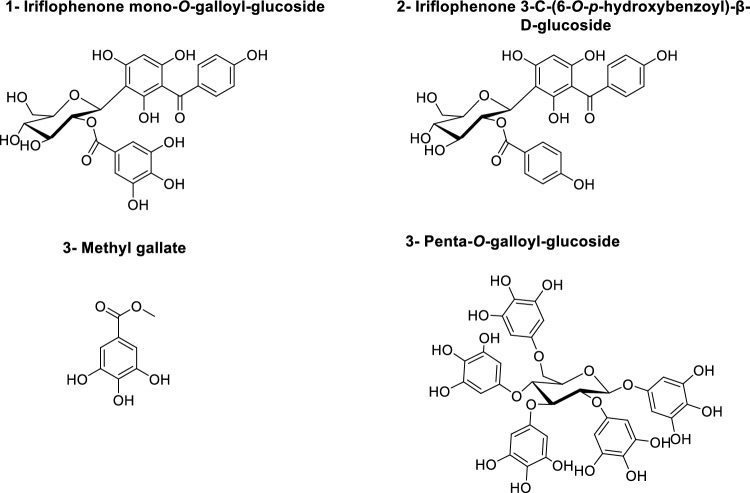
Structures of four isolated compounds from *M. indica.*

### Determination of the QSI activity of *M. indica* isolated compounds

The QSI assay of the four purified compounds isolated from *M. indica* was evaluated by the reporter strain *C. violaceum* ATCC 12472. MG showed potent QSI activity, with inhibition of violacein pigment formation at 33 mm diameter. The other three compounds did not exhibit any QSI activity. As a result, MG was chosen for the assessment of antipathogenic activity against clinical isolates and standard strains of *P. aeruginosa*.

### Minimum inhibitory concentrations

The MICs of MG were 512 µg/mL against PAO1, PA14, Pa21, Pa22, Pa23, Pa24, and PAO-JP2. Therefore, 128 and 256 µg/mL were used as sub-MICs at 1/4 and 1/2, respectively (Table 1).

**Table 1:** MICs and Sub-MICs of MG (1/2 and 1/4).

	MIC (μg/mL)	1/2 MIC (μg/mL)	1/4 MIC (μg/mL)
*P. aeruginosa* Pa21	512	256	128
*P. aeruginosa* Pa22	512	256	128
*P. aeruginosa* Pa23	512	256	128
*P. aeruginosa* Pa24	512	256	128
*P. aeruginosa* PAO1	512	256	128
*P. aeruginosa* PA14	512	256	128
*P. aeruginosa* PAO-JP2	512	256	128

### Effect of MG on the growth of *P. aeruginosa* strains

**Figure 2:**
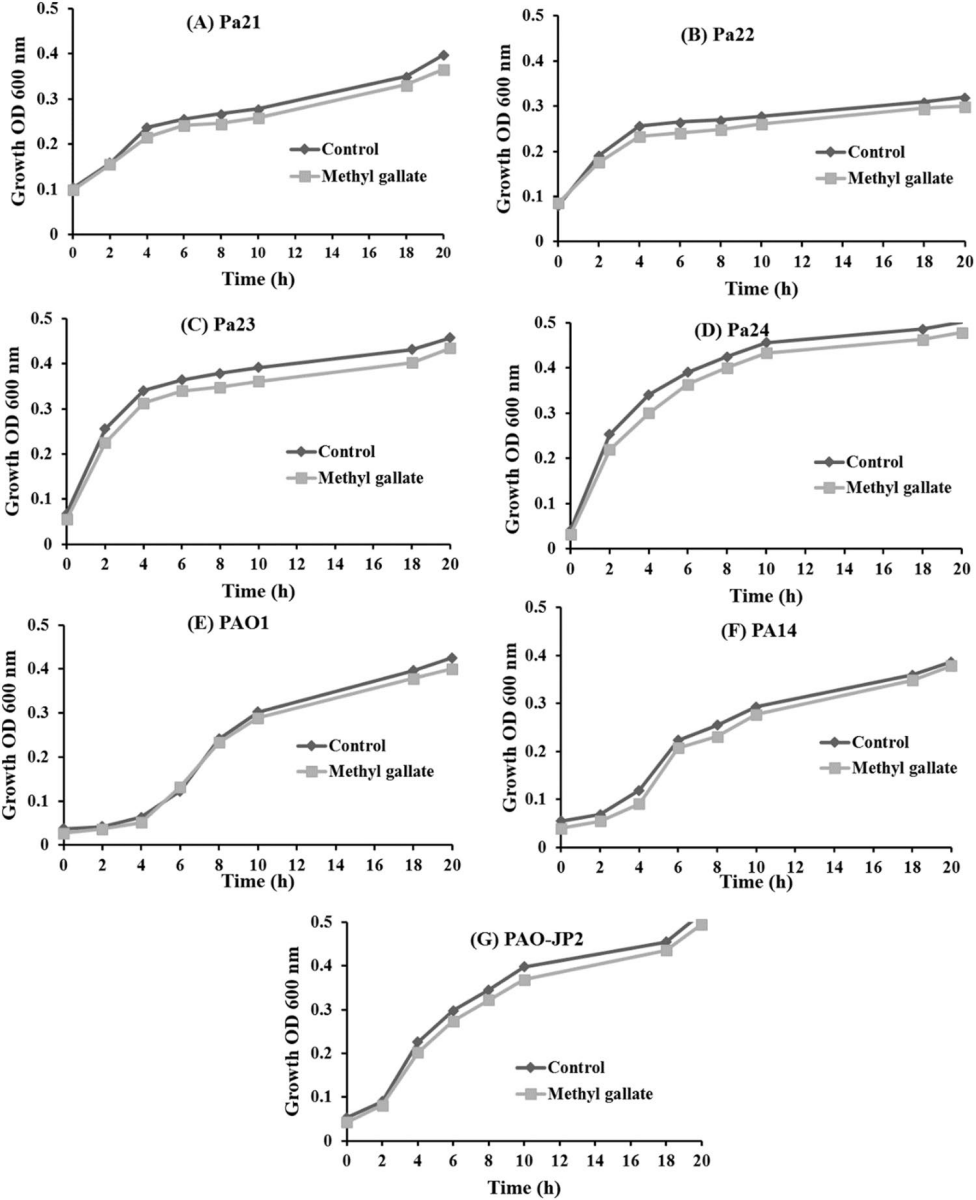
Growth curve of *P. aeruginosa* strains with and without 1/2 MIC of methyl gallate.

The treatment of *P. aeruginosa* strains with 1/2 MIC of MG did not cause any change in growth as compared to the untreated cultures. The bacterial count of untreated cultures was 168, 177, 162, 146, 136, 148, and 187 × 10^7^ CFU/mL for PAO1, PA14, Pa21, Pa22, Pa23, Pa24, and PAO-JP2, respectively. Treated plates contained 160, 172, 157, 145, 130, 139, and 178 × 10^7^ CFU/mL for PAO1, PA14, Pa21, Pa22, Pa23, Pa24, and PAO-JP2, respectively (Fig. 2; supplementary Fig. 23).

### Antivirulence effect of MG on *P. aeruginosa* strains

In comparison with untreated cultures, MG at 1/4 and 1/2 MICs significantly inhibited protease, biofilm, hemolysin, and pyocyanin in Pa21, Pa22, Pa23, Pa24, PAO1, and PA14. Among the examined virulence factors, PAO-JP2 showed the least activity.

### Effect on hemolysin activity

**Figure 3:**
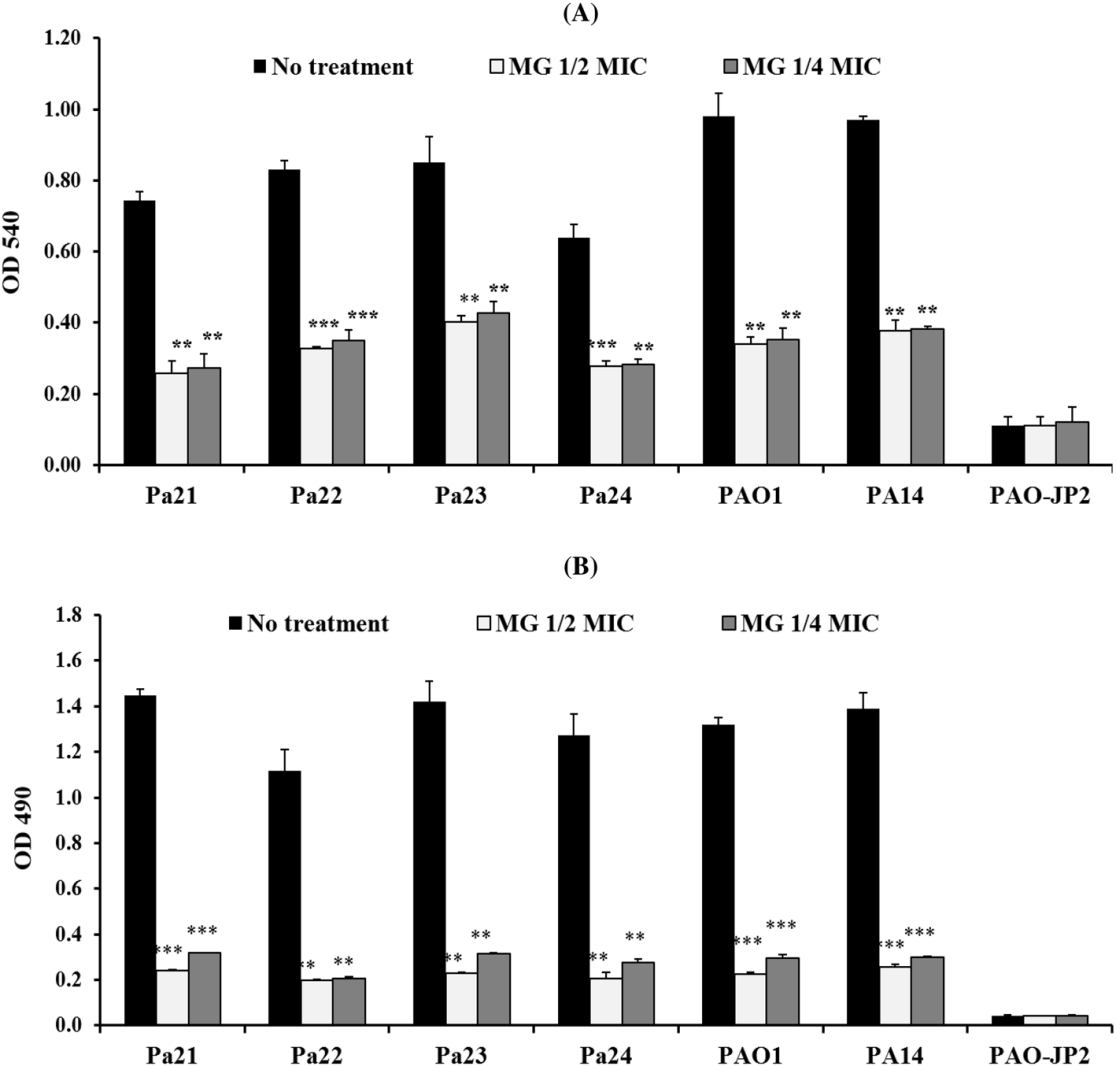
Effect of sub-MICs of methyl gallate on hemolysin activity (**A**) and biofilm formation (**B**) of *P. aeruginosa* strains.

Sub-MICs of MG significantly inhibited hemolysin in Pa21, Pa22, Pa23, Pa24, PAO1, and PA14. As assessed, 1/2 MIC inhibited hemolysin activity by 65.5%, 60.6%, 52.5%, 56.8%, 65.3%, and 61.2%, in strains Pa21, Pa22, Pa23, Pa24, PAO1, and PA14, respectively. In addition, treatment with 1/4 MIC decreased hemolysin by 63.2%, 57.8%, 49.8%, 55.7%, 63.9%, and 60.5% in Pa21, Pa22, Pa23, Pa24, PAO1, and PA14, respectively (Fig. 3A).

### Biofilm assay

Sub-MIC, 1/2 of MG (256 μg/mL) significantly reduced the biofilm formation (*P* ≤ 0.001) in Pa21, Pa22, Pa23, Pa24, PAO1, and PA14 by 83.3%, 82.2%, 83.8%, 83.7%, 83%, and 81.6%, respectively. Biofilm was also significantly reduced with treatment of 1/4 MIC of MG by 77.8%, 81.5%, 77.9%, 78.1%, 77.6%, and 78.6% in Pa21, Pa22, Pa23, Pa24, PAO1, and PA14, respectively (Fig. 3B).

### Total protease production

**Figure 4:**
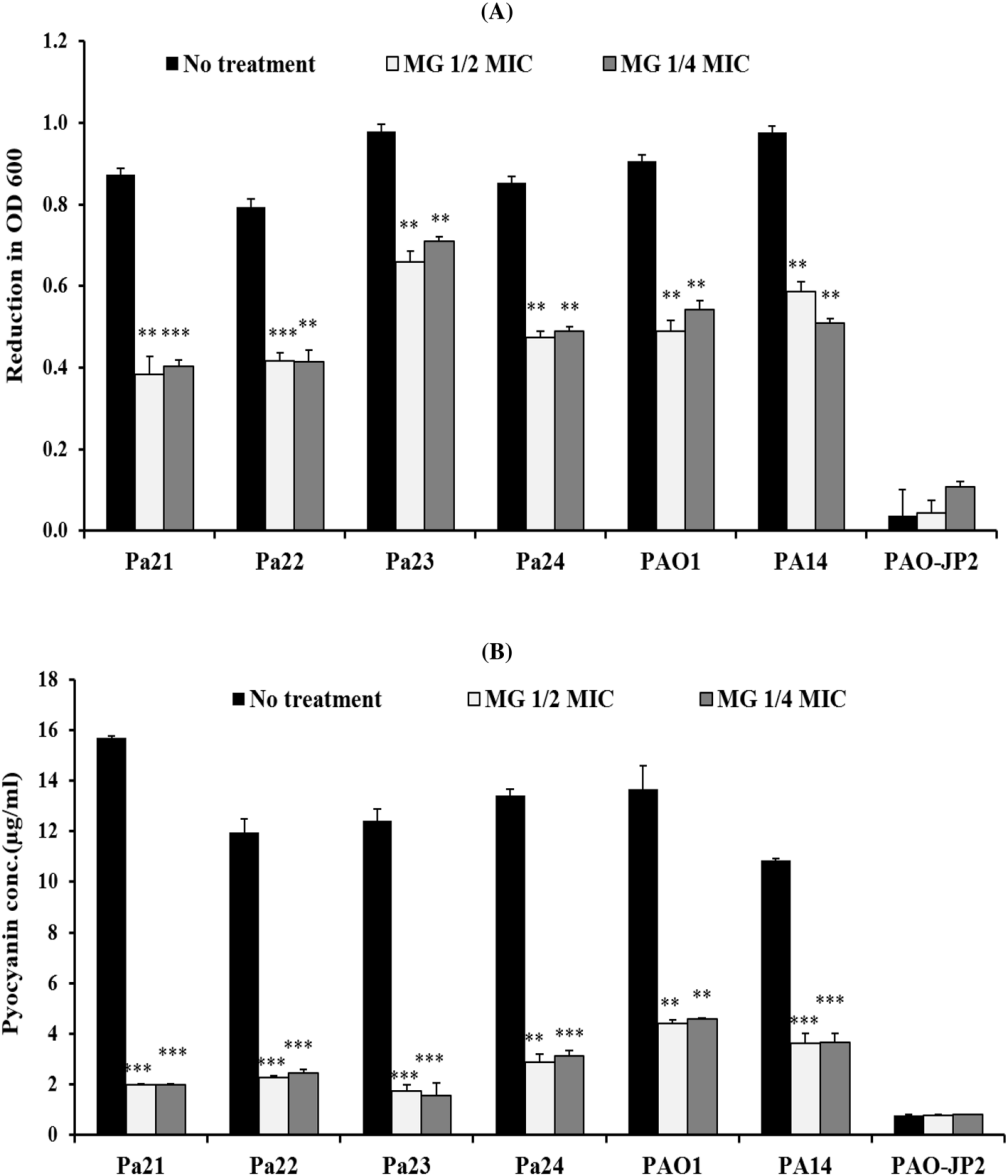
Effect of sub-MICs of methyl gallate on total protease production (**A**) and pyocyanin production (**B**) in *P. aeruginosa* strains.

Protease production in Pa21, Pa22, Pa23, Pa24, PAO1, and PA14 was significantly decreased in cultures treated with 1/2 MIC of MG by 55.9%, 47.3%, 32.7%, 44.3%, 46.2%, and 40.1%, respectively. Similarly, 1/4 MIC of MG significantly lowered the proteolytic activity by 53.6%, 47.6%, 27.6%, 42.4%, 40.3%, and 48% in Pa21, Pa22, Pa23, Pa24, PAO1 and PA14, respectively (Fig. 4A).

### Pyocyanin assay

Pyocyanin was significantly reduced in Pa21, Pa22, Pa23, Pa24, PAO1, and PA14 by MG supplemented at 1/2 and 1/4 sub-MICs (*P* ≤ 0.001). For instance, 1/2 MIC inhibited pyocyanin by 87.3%, 80.9%, 85.9%, 78.7%, 68.1%, and 66.5% in Pa21, Pa22, Pa23, Pa24, PAO1, and PA14, respectively. Meanwhile, 1/4 MIC eliminated the pyocyanin by 87.3%, 79.6%, 87.4%, 76.7%, 66.6%, and 66.3% in Pa21, Pa22, Pa23, Pa24, PAO1, and PA14, respectively (Fig. 4B).

### LasR and ligand binding affinity analysis using molecular docking

**Figure 5:**
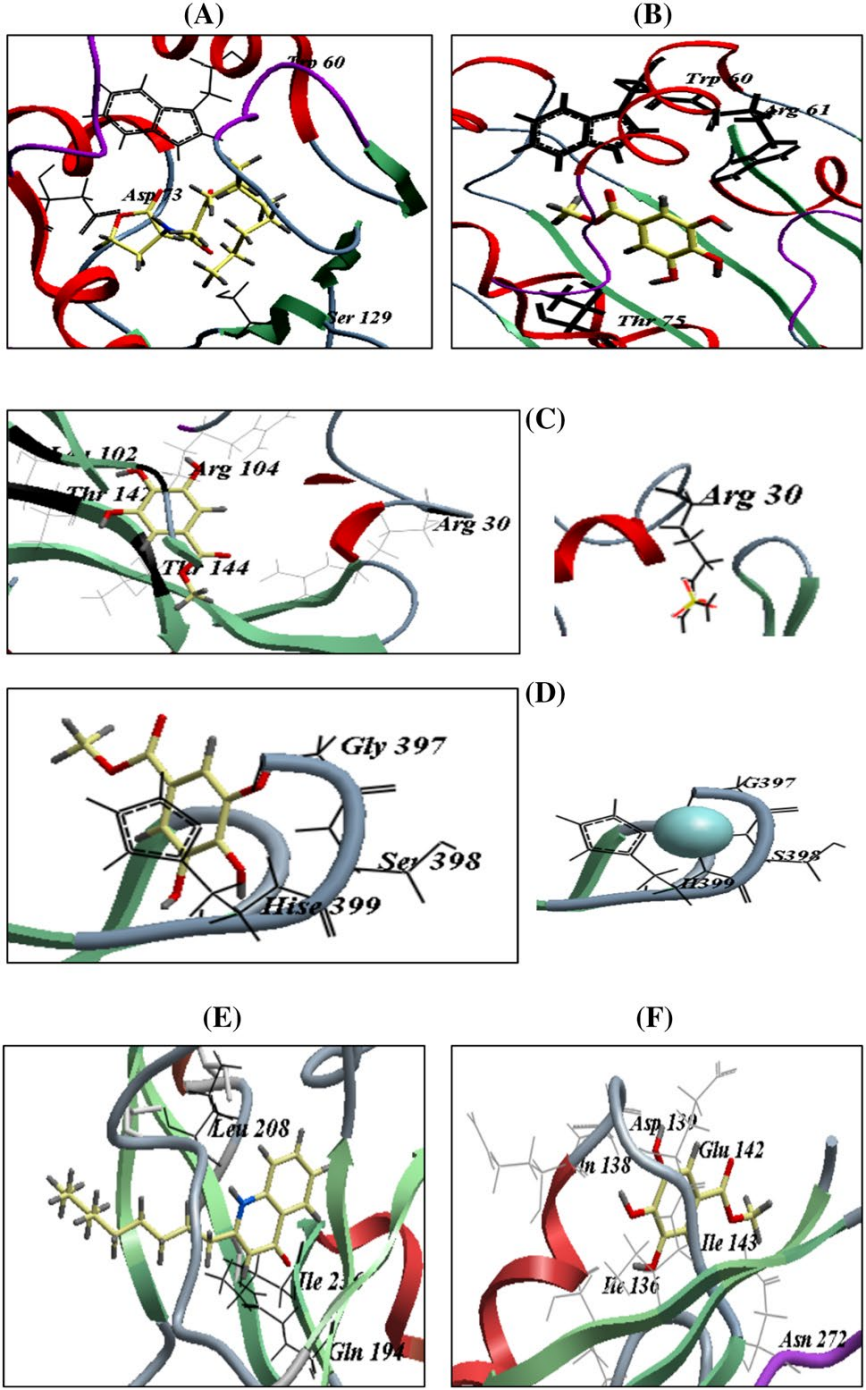
Binding modes of 3-oxo-C12-HSL (**A**) and MG (**B**) into *P. aeruginosa* active site; sulphate ligand (**C**) and MG (**D**) into LasI synthase of *P. aeruginosa*; NHQ ligand (**E**) and MG (**F**) into PqsR coinducer of *P. aeruginosa.*

The autoinducer 3-oxo-C12-HSL was docked at the LasR binding site. As reported previously, this revealed three hydrogen bonds with Ser129, Trp60, and Asp73 and an ICM score of − 107.47. The active site of LasR requires all three of these amino acids. With Trp60, Arg61, and Thr75, MG formed three H-bonds with an ICM score of − 57.18 (Table 2, 3) (Fig. 5A,B).

**Table 2:** Internal coordinate mechanics ratings, the number of H-bonds and amino acid residues.

Receptor	Compound	ICM score	No. of H-bonds	Amino acid residues involved
LasR	3oxo -C_12_-HSL	− 107.47	3	Trp 60, Asp 73, Ser 129
	MG	− 57.18	3	Trp 60, Arg 61, Thr 75
LasI	Sulphate	− 44.03	12	Arg30
	MG	− 50.79	9	Arg 30, Leu 102, Thr 104, Thr 142, and Thr 144
PqsR	NHQ	− 57.78	3	Gln 194, Leu 208, and Ile 236
	MG	− 44.48	6	Ile 136, Gln 138, Asp 139, Glu 142, Ile 143, and Asn 272

**Table 3:** Molecular docking results of methyl gallate with interacting amino acids with LasI of *P. aeruginosa.*

Receptor	Amino acid residues	Atom of amino acid	Atom of compound	length Å
LasR	Trp 60	He1	O4	1.92
	Arg 61	Hh22	O3	2.63
	Thr 75	Og1	H3	2.22
LasI	Arg 30	Hh21	O4	2.09
	Arg 30	Hh22	O4	2.37
	Thr 104	Hn	O1	2.41
	Thr 104	Hn	O2	2.68
	Thr 104	Hh11	O1	2.38
	Thr 142	Hg1	O3	1.86
	Thr 142	Og1	H5	2.36
	Thr 144	Hg1	O5	2.06
	Leu 102	O	H4	1.94
PqsR	Glu 142	Hn	O3	1.62
	Ile 143	Hn	O2	2.56
	Asn 272	Hd22	O5	1.81
	Ile 136	O	H3	1.85
	Gln 138	O	H4	1.89
	Asp 139	O	H5	1.2

### LasI and ligand binding affinity analysis

The original ligand, sulfate, was redocked at the LasI binding site, which had 12 hydrogen bonds with Arg30 and an ICM score of − 44.03. MG had an ICM score of − 50.79 and formed nine H-bonds with Arg 30, Leu102, Thr 104, Thr142, and Thr144 (Table 2) (Fig. 5C). On the other hand, MG also bound to zinc metal active sites and formed H-bonds with Gly397, ser398, and His399 (Fig. 5D).

### PqsR and ligand binding affinity analysis

NHQ redocked into the active site of its receptor, PqsR, which was bound to it by amino acids Gln194, Leu208, and Ile236, with an ICM score of − 57.78. When MG was docked at the active site of PqsR, it had an ICM score of − 44.48 and formed six H-bonds with Ile136, Gln138, Asp139, Glu142, Ile143, and Asn272 (Table 2) (Fig. 5E,F).

## Discussion

*Pseudomonas aeruginosa* is a nosocomial and opportunistic human pathogen that can be isolated from a variety of environments. QS controls pathogenesis by regulating gene expression and the release of virulence factors such as proteases, hemolysin, pyocyanin, and biofilm. The capability of *P. aeruginosa* to create a biofilm that can resist antibiotics has recently made treatment of *Pseudomonas* infections more challenging^28^. Antimicrobial resistance in *P. aeruginosa* is associated with its ability to produce degrading enzymes that can inactivate antimicrobial agents and efflux pumps that have antibiotic resistance genes^29^. As a result, finding new approaches for combating *Pseudomonas* infection is critical; one of these is inhibiting QS and bacterial cross-talk, which can manage bacterial virulence factors.

In this study, the anti-QS activities of several Egyptian medicinal plants were evaluated. *C. violaceum* ATCC 12472 is the most used strain for determining the QSI of compounds by inhibition of its distinctive violet pigment. The production of violacein pigment by reporter strain *C. violaceum* ATCC 12472 is under the control of QS signals (AHL). Therefore, inhibition of violacein pigment without affecting bacterial growth implies potential QSI^30,31^.

The pulp, bark, leaf, fruit peel, heartwood, and seed of *M. indica* have numerous medicinal uses^32^ for treatment of a wide range of human diseases including diarrhea, cough, anemia, gastric disorders, jaundice, malaria, hepatic disorders, hemorrhage, diabetes, and ulcers^33^.

Among the tested extracts, *M. indica* had the greatest ability to inhibit this pigment without any effect on growth. The total extracts of *M. indica* demonstrated potential QSI activity^34^. Hence, fractionation of *M. indica* extract was performed. The highest QSI was produced by a MG compound. Similarly, MG isolated from other plants like *Piper betle* (betel), *Anacardium occidentale* (cashew), and *Anacardium occidentale* L. (cashew) exhibited anti-Qs activity against *C. violaceum* 12472^35,36^. Additionally, MG displayed QSI activity against other pathogenic strains like *Salmonella Typhimurium* alone and in combination with marbofloxacin at 30 μg/mL concentration^37^.

Furthermore, MG is a well-known antibacterial compound and it has been reported to have antibacterial activity against many bacterial strains. For example, it inhibited the growth of plant-pathogenic bacteria *Ralstonia solanacearum*^38^, and some oral bacteria such as *Actinomyces viscosus, Streptococcus mutans,* and *S. sobrinus*^39^*.* Additionally, it inhibited the growth of some *Salmonella* spp. clinical isolates^40^ and it approved a synergistic effect against nalidixic acid-resistant bacteria when combined with nalidixic acid^41^*.* Hence, we tested its effectiveness as QSI on virulence factors of *P. aeruginosa*.

*Pseudomonas aeruginosa* causes many serious nosocomial infections, which are frequently linked to the development of biofilm and are often resistant to the majority of antibacterial drugs^28^. MG inhibited biofilm formation in all tested strains. Therefore, it could be an effective tool for inhibiting biofilm formation and making the biofilm more vulnerable to neutrophil phagocytosis and immune system responses^42^. *P. aeruginosa* produces a green pigment called pyocyanin, which is controlled by the RhlI/R and PQS signaling systems. The removal of this green pigment implies lower levels of pyocyanin content as compared with untreated cultures. Protease and hemolysin are Las-regulated virulence genes in *P. aeruginosa* and are affected by MG to various degrees. They are hydrolytic enzymes that are formed to assist bacteria to infect host tissues and escape the host's defenses^43^. The QSI activity of MG was obtained at sub-minimum inhibitory concentrations of the pure isolated compound (MG) from *M. indica*. As a result, pathogenicity and virulence factors were reduced without affecting viability. Our results are in line with previous work that emphasized the ability of MG to inhibit virulence factors regulated by QS in *P. aeruginosa*^44^.

Similarly, *M. indica* leaf extracts exhibit QSI with a decrease in total protease production of 43.8–56% and a 72% reduction in biofilm formation in *P. aeruginosa* at sub-MIC 800 mg/mL^34^. Furthermore, MG has been reported to disrupt biofilm formation in *P. aeruginosa* by 70%^45^ and in *S. mutans*^46^*.* Also, MG was reported to inhibit pyocyanin production in *P. aeruginosa* PAO1 by 80%^44^*,* by 65% at the concentration of 12.5 μg/mL after 18 h of incubation^47^, by 64% at the concentration of 256 μg/mL^45^. Additionally, MG could eliminate protease production in *P. aeruginosa* by 51% at 256 μg/mL^45^ and Gallic acid also eliminates protease production in *P. aeruginosa*^48^.

Hence, utilization of natural compound MG with elimination of QS-related virulence factors could be an appropriate alternative therapy for inhibition of microbial dissemination without development of microbial resistance.

On a structural basis, virtual screening and protein docking against LasR receptor was performed by using Molsoft ICM 3.4-8C software to determine the QS inhibitory potential of the MG compound^49^. The Protein Data Bank was used to obtain the PDB structure of the receptor protein LasR (PDB ID: 2UV0). Calculating the scoring and hydrogen bonds with the surrounding amino acids at the active site of LasR revealed the binding mode, affinity, and orientation of MG. According to PDBsum, the amino acids at the LasR active site (PDB ID: 2UV0) were Trp60, Asp73, Cys79, Tyr64, Gly126, Ala50, Trp88, Tyr56, Thr75, Tyr93, Ala105, Leu110, and Ser129. The polar groups of AHL and the residues Asp73, Trp60, Tyr56, and Ser129 of LasR form H-bond interactions that are responsible for the correct folding of the LuxR type of protein^30,50,51^. It has been reported that 3oxo-C_12_-HSL forms three H-bonds with the amino acids Asp73, Trp60, and Tyr56^31^. In agreement with the MG docking results, many H-bonds are formed between LasR and the HSL of autoinducer 3-oxo-C_12_-HSL, such as Ser 129, Tyr 56, Trp 60, Arg 61, Asp 73, and Thr 75, which is similar to findings on pyridoxal lactohydrazone^52^. Surface rendering of LasI revealed that the tunnel in the acyl-chain binding region is formed by the following residues: Trp33, Trp69, Met79, Leu102, Phe105, Thr121, Leu122, Met125, Leu140, Thr142, Thr144, Val148, Met151, Met152, Ala155, Leu157, Ile178, and Leu188. Several of these residues, including Met79, Phe105, Thr142, and Thr144, are well-conserved among AHL-synthases^53^. MG bound to Arg30 sulfate, the ligand of the enzyme, and leu102, Arg104, Thr 142, and Thr144, well-conserved amino acids. Moreover, MG also bound to a zinc ion binding site in the enzyme. Hence, it acts as a competitive inhibitor by a dual mechanism, either competing with sulfate for its binding site or binding to Gly397, ser398, and His399 amino acids to inhibit the catalytic role of the Zn ion into LasI enzyme. On the other hand, NHQ redocked into the PqsR active site had an ICM score of − 57.78 and formed H-bonds with Gln194, Leu208, and Ile236, which are amino acids at the active sites of the PqsR enzyme^54^. When MG was docked into the active site of PqsR, it had an ICM score of − 44.48 and formed H-bonds with amino acids Ile136, Gln138, Asp139, Glu142, Ile143, and Asn272. None of these were reported as amino acids at the active site, which suggests that the activity of MG is due to its binding with LasR and LasI rather than PqsR.

## Conclusions

This study demonstrated that MG purified from *M. indica* medicinal plants leaves significantly reduced the pathogenicity and virulence factors including elastase, protease, pyocyanin production, hemolysin and distrusted biofilm of *P. aeruginosa* without affecting its growth rate for the first time. This could play a critical role in combating MDR, which has been identified by WHO as one of the top 10 global public health risks especially in immune-compromised patients.

## Materials and methods

### Bacterial strains, culture conditions, and media

The reporter strain *Chromobacterium violaceum* ATCC 12472^55^ was used to screen the QSI activity of the natural extracts. The strain was inoculated on Luria–Bertani (LB) media containing 1% (w/v) tryptone, 0.5% (w/v) yeast extract, and 1% (w/v) NaCl at pH 7, solidified with 2% (w/v) agar, and grown overnight at 28 °C for 48 h^56^. The clinical isolates of *P. aeruginosa*, Pa21, Pa22, Pa23, and Pa24, were obtained from urine samples according to the ethical committee standards of the Faculty of Medicine, Alexandria University, Egypt (Institutional Review Board (IRB), No. 0201472, 18 March 2021, Faculty of Medicine, Alexandria University, Egypt). The identification of *P. aeruginosa* was based on laboratory biochemical standards^57^.

The bacterial isolates Pa21, Pa22, Pa23, and Pa24 identified as *P. aeruginosa* (collection number QSCC6422 as Quorum Sensing Culture Collection, Mansoura University, Egypt). Standard strains including *P. aeruginosa* PA14 (DSM 19882), *P. aeruginosa* PAO1 (ATCC47085), are kindly provided by Prof. Keller, UW, USA^58^, and the QS-negative control strain *P. aeruginosa* PAO-JP2, are kindly provided by Prof. Martin Schuster, Department of Microbiology, Oregon State University, Nash Hall, Corvallis, OR 97331^59^. All *P. aeruginosa* strains were grown in LB broth media and kept in an incubator at 37 °C overnight.

### Plant crude extracts

Fresh leaves of *Mangifera indica* L. were collected from Mansoura University gardens (permitted for researchers) in May 2017 and complied with institutional guidelines and legislation. The plant was verified by staff members of the Pomology Department, Faculty of Agriculture, Mansoura University. Aerial parts of *Atriplex halimus* L., *Euphorbia paralias* L., *Retama raetam* (Forssk.) Webb & Berthel., *Eichhornia azurea,* and *Pituranthos tortuosus* (Desf.) Benth. & Hook.f. ex Asch. & Schweinf. plants were collected from Borg El-Arab, Egypt in November 2016 after the permit of the landowners and according to institutional guidelines and legislation. The plants were identified by the staff members of the Botany and Microbiology Department, Alexandria University, Alexandria (Prof. Salama El-Darier Professor of botany, Botany and Microbiology Department, Alexandria University, Alexandria and Prof. Sania Kamal, Professor of botany, Botany and Microbiology Department, Alexandria University, Alexandria).

Voucher samples of the plants were preserved under the codes MI-L-17, AH-A-16, EP-A-16, RR-A-16, EA-A-16, and PT-A-16 in the Herbarium of Pharmacognosy Department, Faculty of Pharmacy. The plants were rinsed in tap water, allowed to dry in the shade for six weeks, and then pulverized into a fine powder. Each dry powder (50 g) was extracted with 70% ethanol and incubated overnight at 30 °C and 200 rpm. The plants were filtered, and extracts were concentrated using a rotary evaporator at 40 °C under vacuum.

### QSI assay of crude extracts

The LB agar plates were prepared, and after solidification, they were overlaid with soft LB medium (1% w/v agar) inoculated with *C. violaceum* ATCC 12472. A sterile cork borer was used to carve out wells (10 mm diameter) in the double-layered plates. A 100 µL aliquot of the concentrated plant ethanolic extract was added to the corresponding wells. Plates were incubated at 28 °C for 48 h, and the inhibition of violacein pigment around each well was measured^55^. In the assay plates, a well with ethanol was used as a negative control.

### Isolation of the active fraction of *M. indica*

The shade-dried leaves of *M. indica*, which showed the highest QSI activity, were pulverized to a powder (940 g). Next, 4 L of ethanol was used to extract the entire amount of dry powder for 48 h at room temperature, until it was completely used up. The combined ethanol extracts were dried in a vacuum using a rotary evaporator at 40 °C to yield 69.3 g of crude extract. The extract was dissolved in 300 mL water and divided in a polarity-based manner with petroleum ether, ethyl acetate, methylene chloride, and butanol. The fractions were collected, dried, and weighed (21.7, 18.9, and 19 g, respectively).

A total of 3.3 g ethyl acetate extract was added to VLC of RP-18 silica (20 cm × 4 cm) and isocratically eluted with a mobile phase comprised of H_2_O-MeOH (7:3) to collect 25 fractions (MI-1-25). Fraction MI-4 (137 mg) was applied to normal phase silica gel and eluted with the gradients H_2_O:CH_3_OH:CHCl_3_:EtOAc (1:4:6:10, then 1:4:6:6). The collected column elites were examined by TLC, and similar fractions were combined, resulting in the purification of compound **1** (22 mg). Similarly, fraction MI-9 (187.2 mg) was chromatographed to yield compound **2** (17 mg). Fractions MI-5 (112 mg) and MI-11 (131 mg) were applied separately to the top of a glass column of normal silica gel and the gradients H_2_O:CH_3_OH:CHCl_3_:EtOAc (1:4:8:15, 1:4:6:10, and 1:4:6:6, respectively) were used as a mobile phase to elute them. The columns resulted in the isolation of compounds **3** (16 mg) and **4** (19 mg), from fractions MI-5 and MI-9, respectively.

### Characterization of isolated compounds by NMR spectroscopy

Before biological evaluation, NMR experiments (1D and 2D) were performed on a Varian Dual Broadband Probe 400 MHz, Bruker Avance III 600, or Bruker DRX-500 and 400 MHz spectrometer using pyridine-*d*_5_, CD_3_OD, or DMSO-*d*_6_ as solvents to identify the isolated compounds. The internal index for modification was the solvent peaks. On a mass spectrometer (Agilent Technologies 6200 series), negative and positive ion modes of mass spectra were observed. To measure the precise rotations, an automatic polarimeter IV was used. UV spectra were recorded by a Varian Cary 50 Bio UV–visible spectrophotometer. Column chromatography was performed using a flash silica gel and reversed-phase C-18. Analytical TLC was performed on a silica gel F254 aluminum sheet (20 × 20 cm, Fluka) or a Silica 60 RP-18 F254S aluminum sheet (20 × 20 cm, Merck), and detection was performed using UV-254 nm. Next, 1% vanillin in concentrated H_2_SO_4_-EtOH (10:90) was sprayed and then heated with a heat gun to visualize the spots. Analytical-grade solvents (Fischer Chemicals) were used in the purification and isolation procedures.

### Determination of the QSI activity of the purified compounds

Four isolated compounds from *M. indica* were dissolved in DMSO and tested for their QSI activity using *C. violaceum* ATCC 12472. Each well (10 mm diameter) was filled with 50 µL of each compound. The inhibition of the violet zone around the well of induced violacein production was measured in millimeters. DMSO was used as the control.

### Determination of minimum inhibitory concentrations

Using the microtiter plate assay method, the minimum inhibitory concentration (MIC) of methyl gallate (MG) was determined. MH broth was distributed at 100 µL in each well. The MG compound was serially diluted 1:1 in 10 dilutions (0.5–512 µg/mL). *P. aeruginosa* cultures PAO1, PA14, Pa21, Pa22, Pa23, Pa24, and PAO-JP2 were added to each well to a final concentration of 1 × 10^5^ CFU/well^60^. Wells containing media alone were considered as the negative control, while wells containing media inoculated with each of the tested strains were considered as the positive control. Microbial growth was detected in each well visually after overnight incubation and compared with the positive control^60^.

### Effect on bacterial growth

*Pseudomonas aeruginosa* strains that were untreated or treated with MG at 1/2 MIC (256 µg/mL) were incubated overnight at 37 °C. Treated and untreated samples (1 mL) were taken every hour and diluted at 1:10 for bacterial counting. Using the pour plate method, the vitality of the treated *P. aeruginosa* samples was determined and compared to the untreated culture. Each diluted sample was added to melted nutrient agar medium (20 mL) at 50 °C, mixed, and poured into 9 cm sterile plates. The plates were incubated overnight at 37 °C, and the colonies were counted. The growth curves of *P. aeruginosa* (treated and untreated) were estimated^61,62^.

### Effects of MG sub-MICs on *Pseudomonas* virulence factors

*Pseudomonas aeruginosa* clinical isolates Pa21, Pa22, Pa23, and Pa24 were treated with 1/4 and 1/2 MICs of MG. Cultures with and without MG were incubated overnight at 37 °C. Cells were removed by centrifugation, and the cell-free supernatant was used for the detection of *Pseudomonas* virulence factors (hemolysin, elastase, protease, and biofilm) in both untreated and treated cultures in triplicate^62,63^. The same conditions were used to evaluate the standard strains PAO-JP2, PA14, and PAO1^64^.

### Determination of hemolysin activity

The hemolysin test of treated and untreated *P. aeruginosa* supernatants was performed by mixing bacteria supernatants with washed RBC suspension (2% v/v)^65^. The mixture of 500 of cell-free supernatant was mixed with 700 µL erythrocytes. The mixture was incubated at 37 °C for 2 h and then centrifuged at 4000 rpm for 10 min at 4 °C. The absorbance of the supernatant at OD_540_ nm, which indicates the level of hemolysin activity, was measured^66^.

### Biofilm assay

The development of biofilm by *P. aeruginosa* strains was assessed using the microtiter plate crystal violet staining method. Wells were inoculated with 100 µL of treated and untreated cultures, and the plates were incubated for 24 h at 37 °C. The wells were rinsed with saline and set for 15 min in 150 µL of absolute methanol. Crystal violet (1% w/v) was used to stain the bacterial cells, and any excess stain was removed by washing the plate with water. The plates were then allowed to air dry. A volume of 150 µL of glacial acetic acid; 33% (v/v) was used to dissolve the dye that bound to the formed biofilm, and the absorbance was determined at OD_490_^67^.

### Determination of protease production

The total protease production of *P. aeruginosa* cultures with or without 1/4 and 1/2 MICs of MG was assessed using the skim milk method^68^. The assay was carried out by mixing 500 µL of supernatants with 1 mL of 1.25% (w/v) skim milk. The mixture was incubated for 1 h at 37 °C, and the absorbance was determined at OD_600_ nm. A reduction in OD_600_ indicated that the skim milk was cleared and that proteolysis activity had increased^69^.

### Pyocyanin assay

Pyocyanin pigment production was assessed by the cultivation of *P. aeruginosa* strains in King A broth media (MgCl_2_ 0.14% (w/v); peptone, 2% (w/v), and K_2_SO_4 _,  1.0% (w/v)) with or without 1/4 and 1/2 MICs of MG^70^, and incubated at 37 °C for 48 h at 200 rpm. The cultures were centrifuged at 4 °C for 10 min at 3000 rpm, and chloroform was used to extract the pyocyanin. The mixture was vortexed after the addition of chloroform until the color turned greenish-blue and then centrifuged for 10 min at 3000 rpm. The chloroform layer was separated and a volume of 1 mL of 0.2 M HCL was added to the mixture with the development of pink color. The absorbance was evaluated at OD_520_ nm. Pyocyanin concentration (μg/mL) was calculated by multiplying the OD_520_ nm by 17.072^70,71^.

### Molecular docking

To determine the binding affinities, binding modes, and inhibition behavior at the LasR active site, MG was docked into the binding site of three QS systems in *P. aeruginosa*: LasR (PDB ID: 2UV0)^72^, LasI (PDB ID: 1RO5)^53^, and PqsR (PDB ID: 4JVD)^54^ to evaluate their binding affinities, inhibitory activities, and binding modes. The crystal structure was obtained using the protein data bank. Many of the protein's water ligands were extracted, and the components were created on ChemBioDraw using ChemOffice (PerkinElmer Informatics). The energy was reduced using MM2. PDB file 2UV0 was then transformed into an internal coordinate mechanics (ICM) object using Molsoft software to conduct docking experiments. ICM were as described by^49^. ICM seeks to identify the global minimum energy that most accurately characterizes the ligand-receptor interaction. The autoinducer molecule 3oxo-C12-HSL interacted with 2UV0 as a typical docked model.

### Data analysis and statistics

The standard deviations and means of the three different measurements were computed using an Excel data sheet, and the experiments were performed in triplicate. Differences between strains that were untreated or treated with MG were considered significant when the probability value was *P ≤ 0.05, **P ≤ 0.01, or ***P ≤ 0.001. The results of the statistical analysis were evaluated by Welch’s *t*-test.

### Ethical approval and consent to participate

Institutional Review Board (IRB), No. 0201472, Faculty of Medicine, Alexandria University, Egypt, assessed this study and found it to be exempt on March 18, 2021.

### Supplementary Information

Supplementary Information.

## Data Availability

The original contributions that were made and presented in the article and in the supplementary material. The corresponding authors can be contacted for more information.
