# Supplementary material for: Inhibition of Pseudomonas aeruginosa quorum sensing by methyl gallate from Mangifera indica
Source: Sci Rep. 2023 Oct 20;13:17942. doi: 10.1038/s41598-023-44063-0 (PMC10589227; doi:10.1038/s41598-023-44063-0)
Supplement: Supplementary file 1 — Supplementary Information. [file 41598_2023_44063_MOESM1_ESM.pdf]

Inhibition of *Pseudomonas aeruginosa* Quorum Sensing by a Methyl Gallate Compound from *Mangifera indica*

Nourhan G. Naga[1], Ahmed A. Zaki [2], Dalia E. El-Badan[1], Heba S. Rateb[3], Khalid M. Ghanem[1], Mona I. Shaaban[4]

[1]Botany and Microbiology Department, Faculty of Science, Alexandria University, Egypt,

[2]Pharmacognosy Department, Faculty of Pharmacy, Mansoura University, Egypt.

[3]Department of Pharmaceutical and Medicinal Chemistry, Faculty of Pharmacy, Misr University for Science and Technology, Cairo, Egypt

[4]Microbiology and Immunology Department, Faculty of Pharmacy, Mansoura University, El Mansoura, Egypt

Corresponding author: Mona I. Shaaban

Phone: +201092535893; fax: (+2) 050-2246253

E-mail: mona_ibrahem@mans.edu.eg, sarafawzy2002@yahoo.com

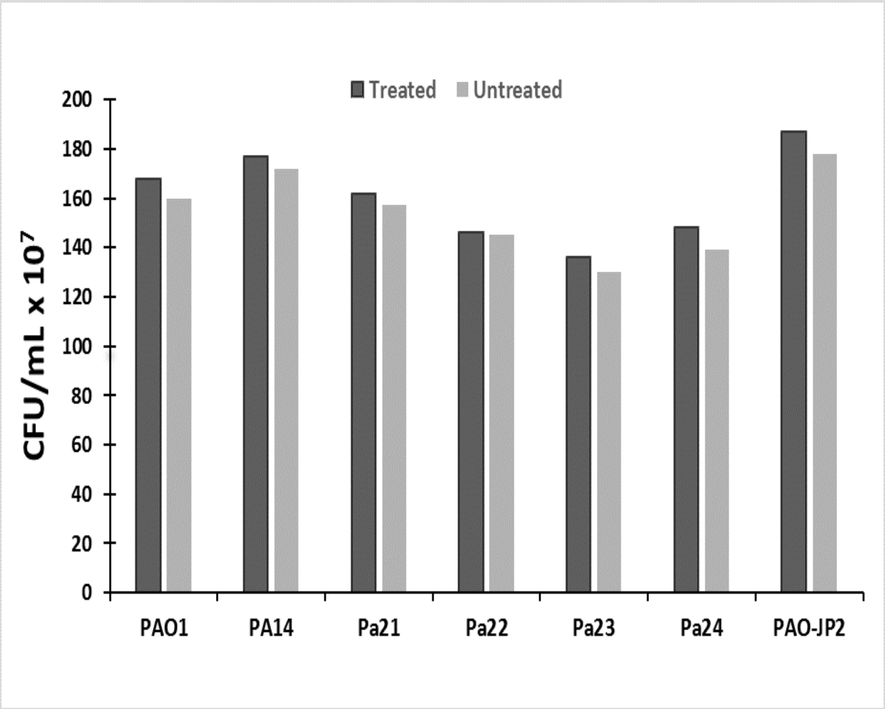

Treated
Untreated
CFU/mL x 10^7
200
180
160
140
120
100
80
60
40
20
0
PAO1
PA14
Pa21
Pa22
Pa23
Pa24
PAO-JP2

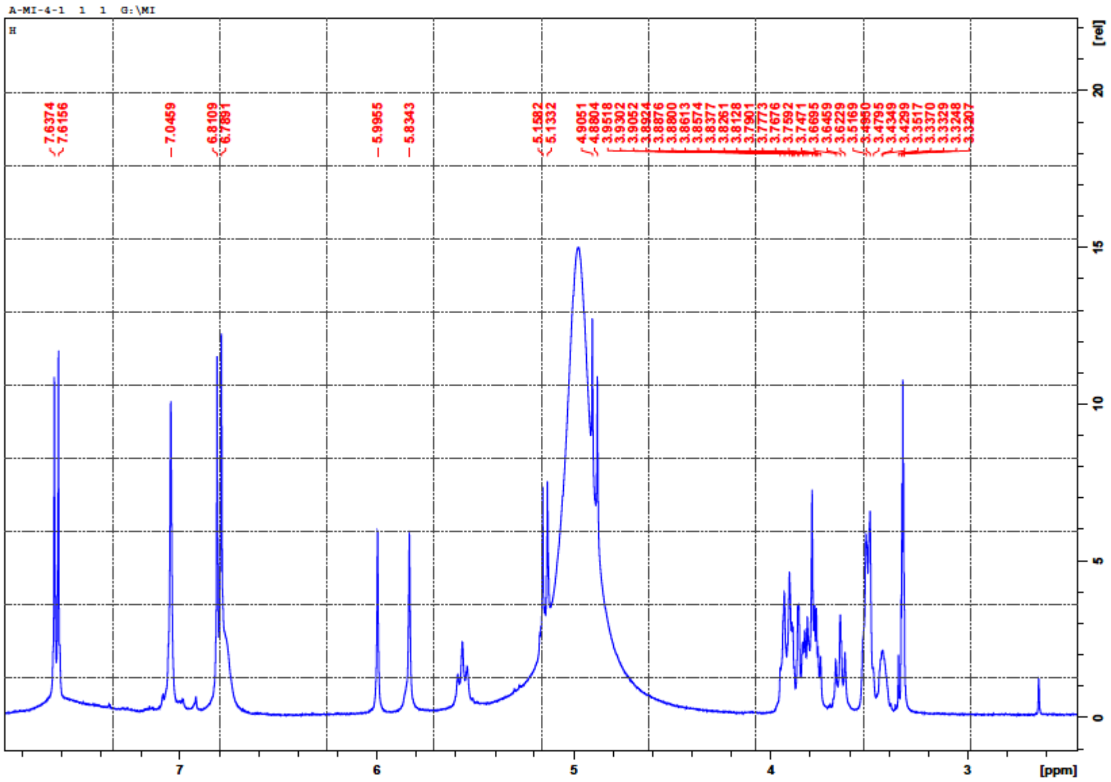

Supplementary figure S1: ^{1}H-NMR Spectrum of Compound **4-1** (400 MHz, CD3OD)

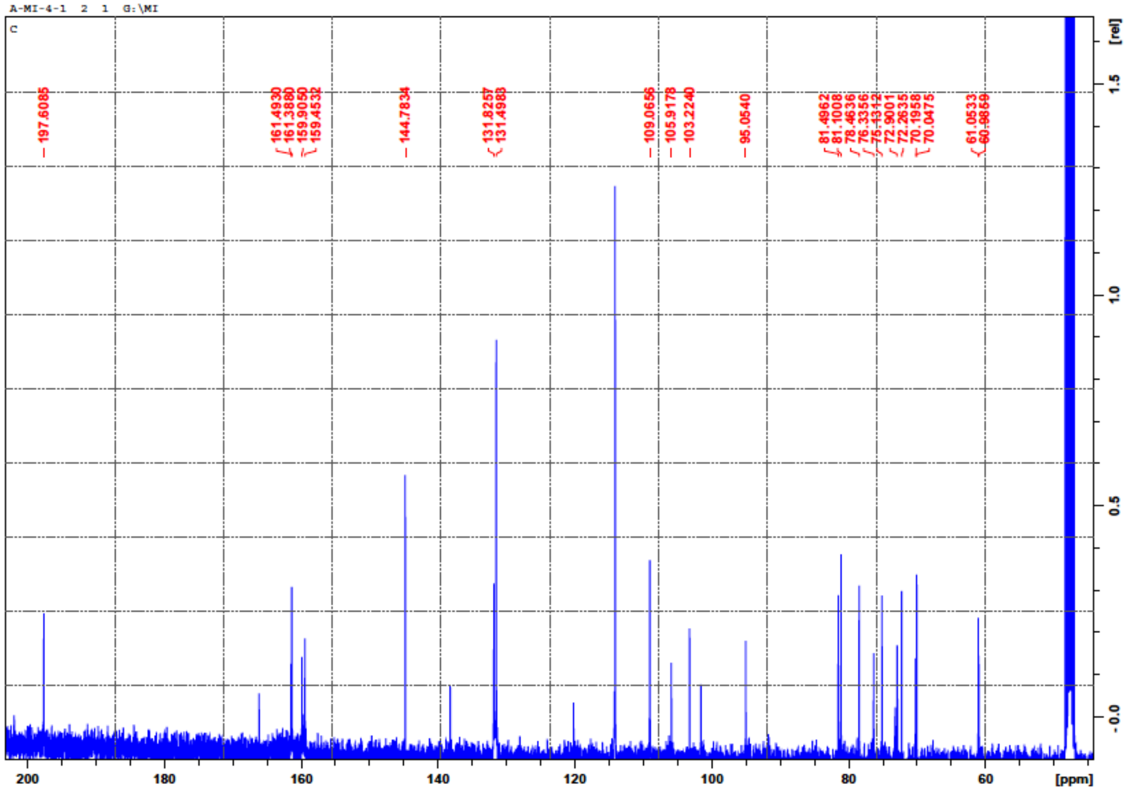

Supplementary figure S2: ^{13}C-NMR Spectrum of Compound **4-1** (100 MHz, CD3OD)

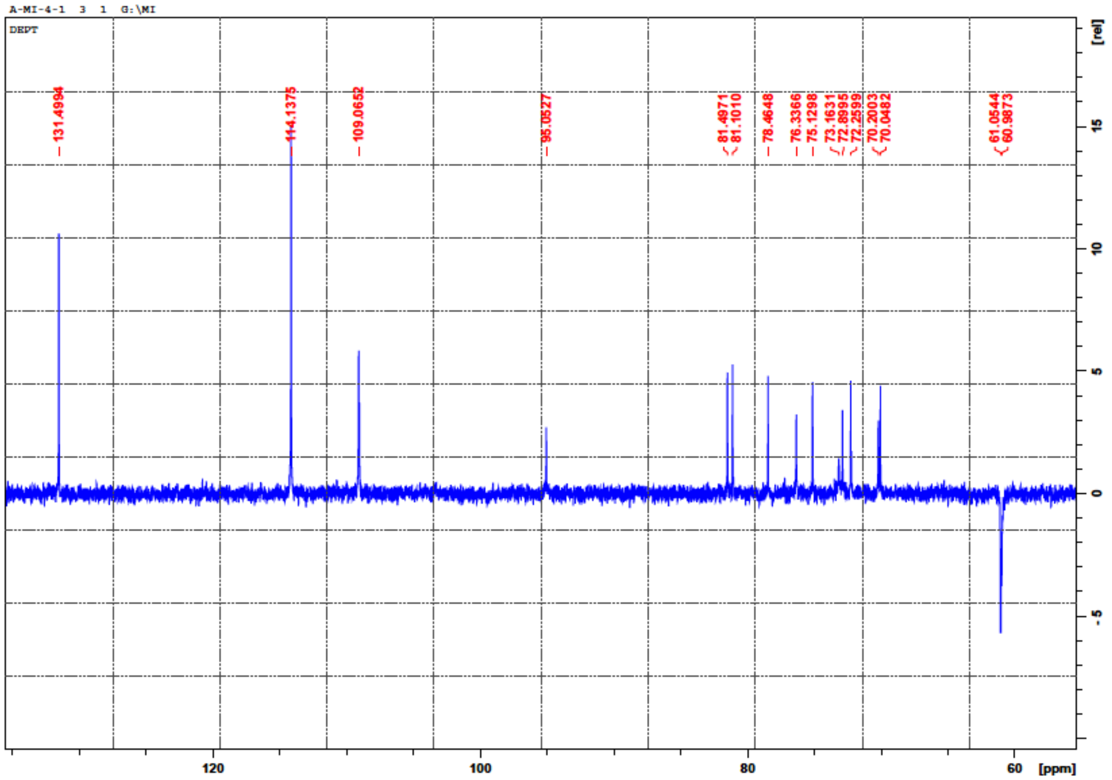

Supplementary figure S3: DEPT Spectrum of Compound **4-1** in CD3OD

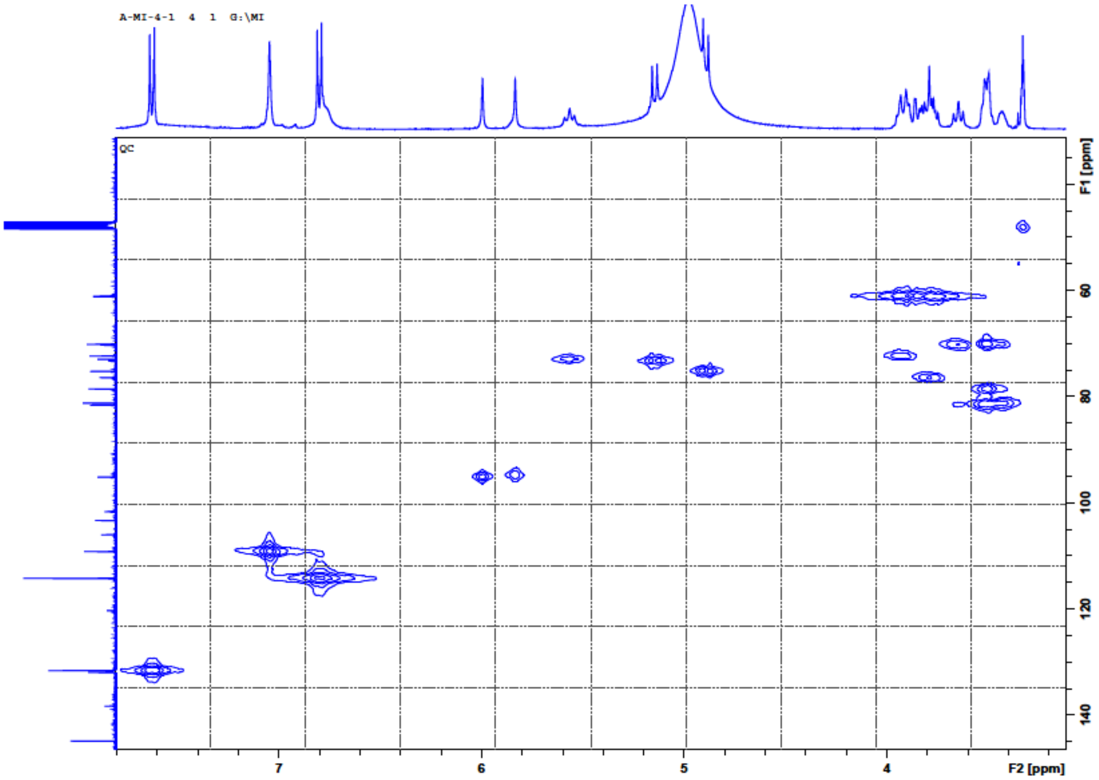

Supplementary figure S4: HMQC Spectrum of Compound **4-1** (400 MHz, CD3OD)

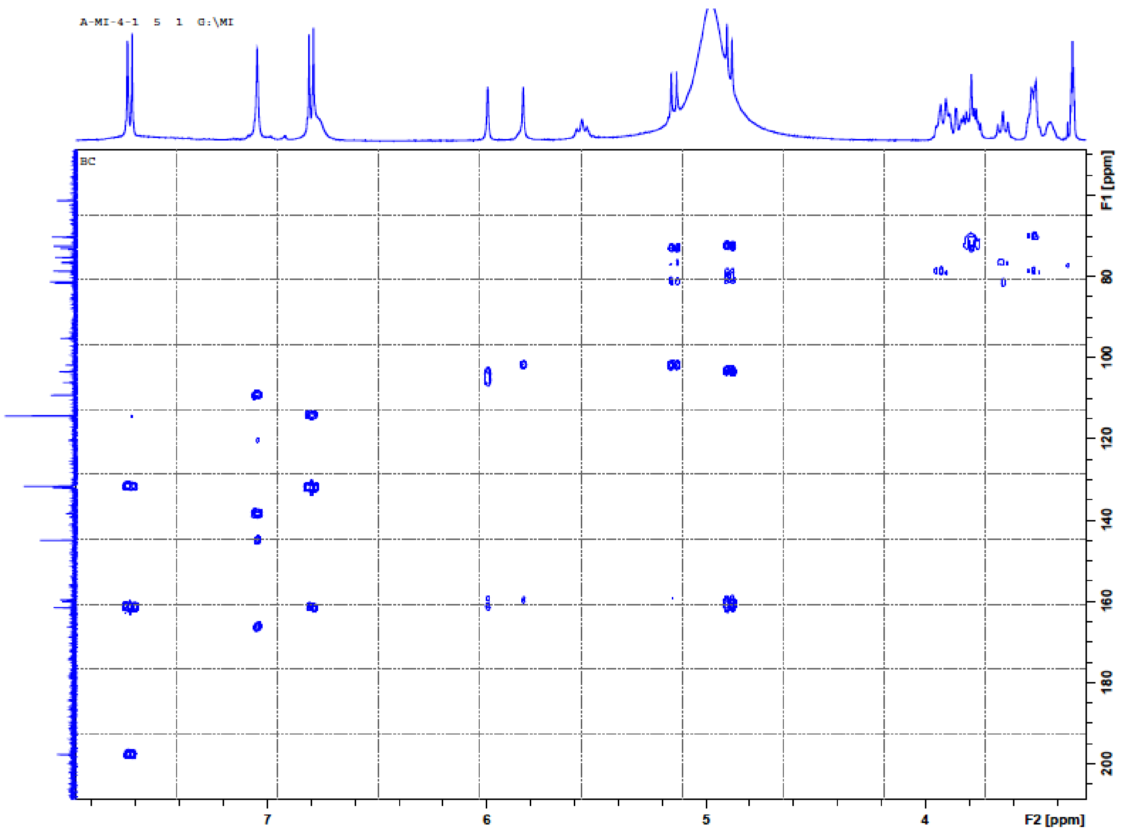

Supplementary figure S5: HMBC Spectrum of Compound **4-1** (400 MHz, CD3OD)

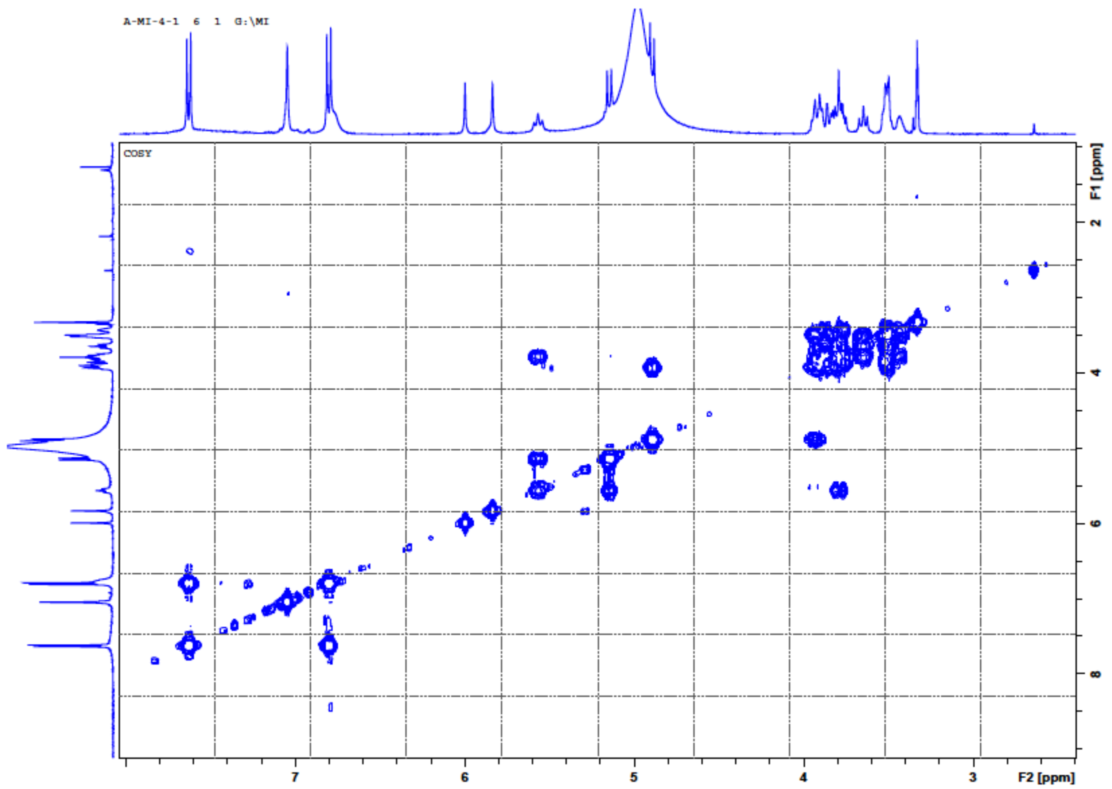

Supplementary figure S6: COSY Spectrum of Compound **4-1** (400 MHz, CD3OD)

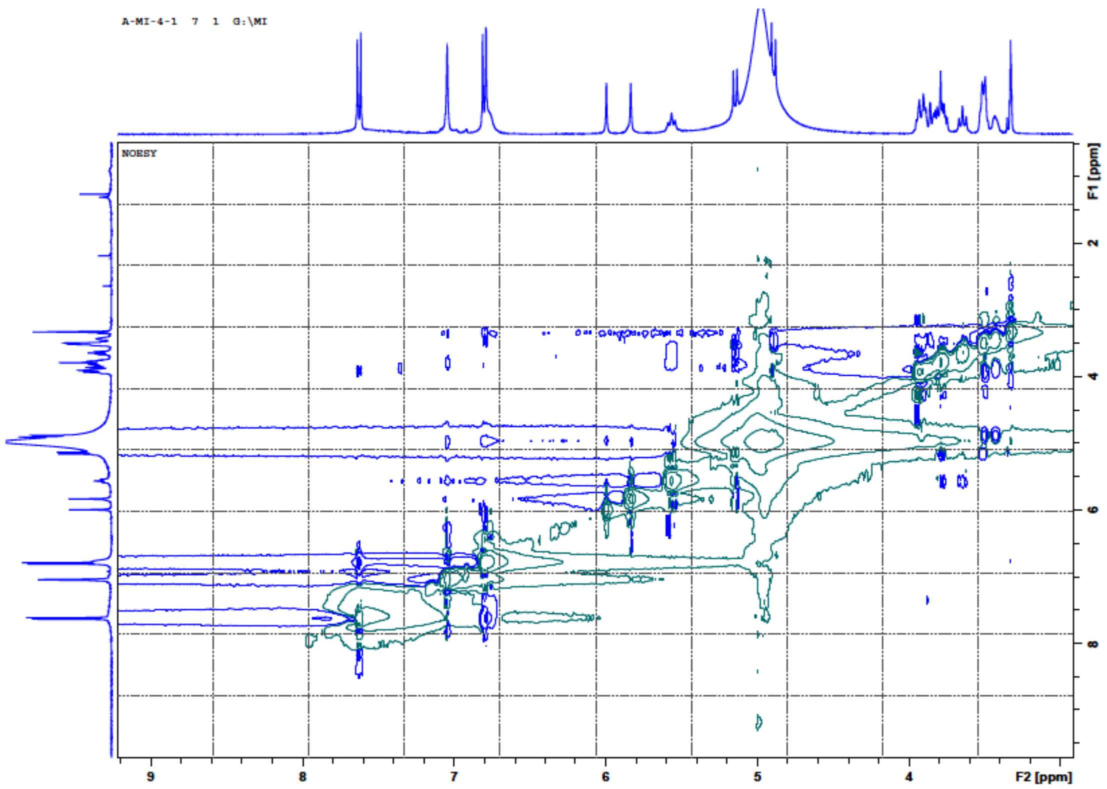

Supplementary figure S7: NOESY Spectrum of Compound **4-1** (400 MHz, CD3OD)

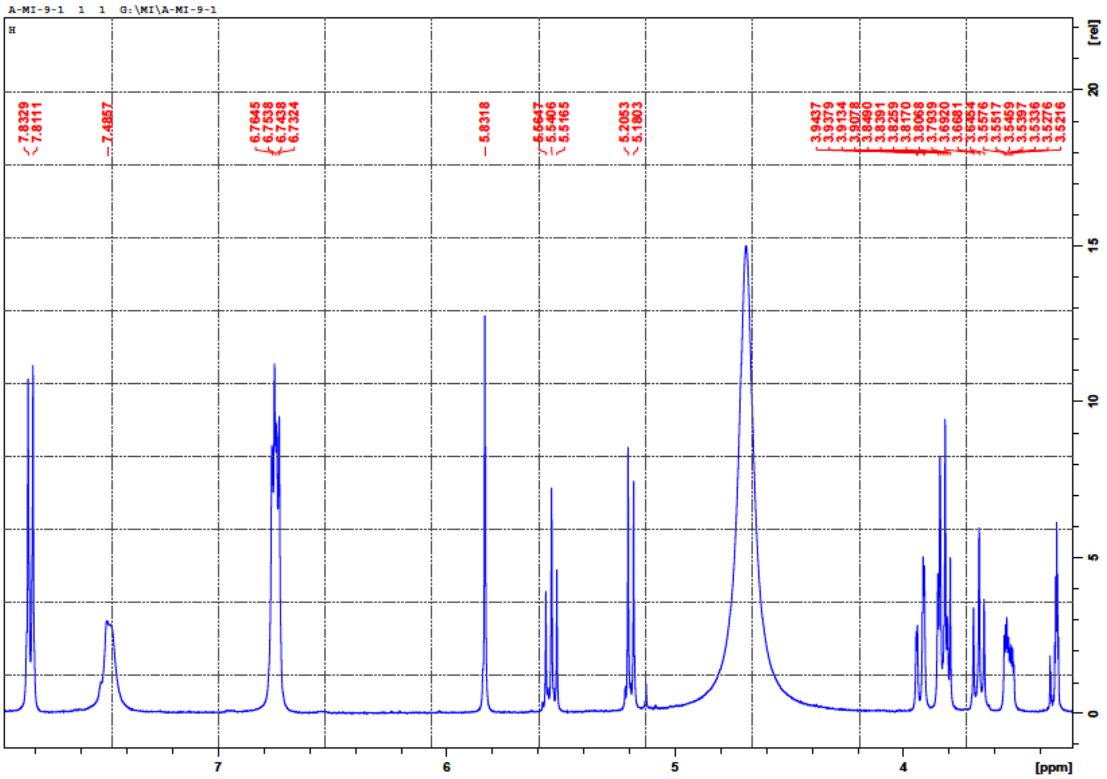

Supplementary figure S8: ^{1}H-NMR Spectrum of Compound **9-1** (400 MHz, CD3OD)

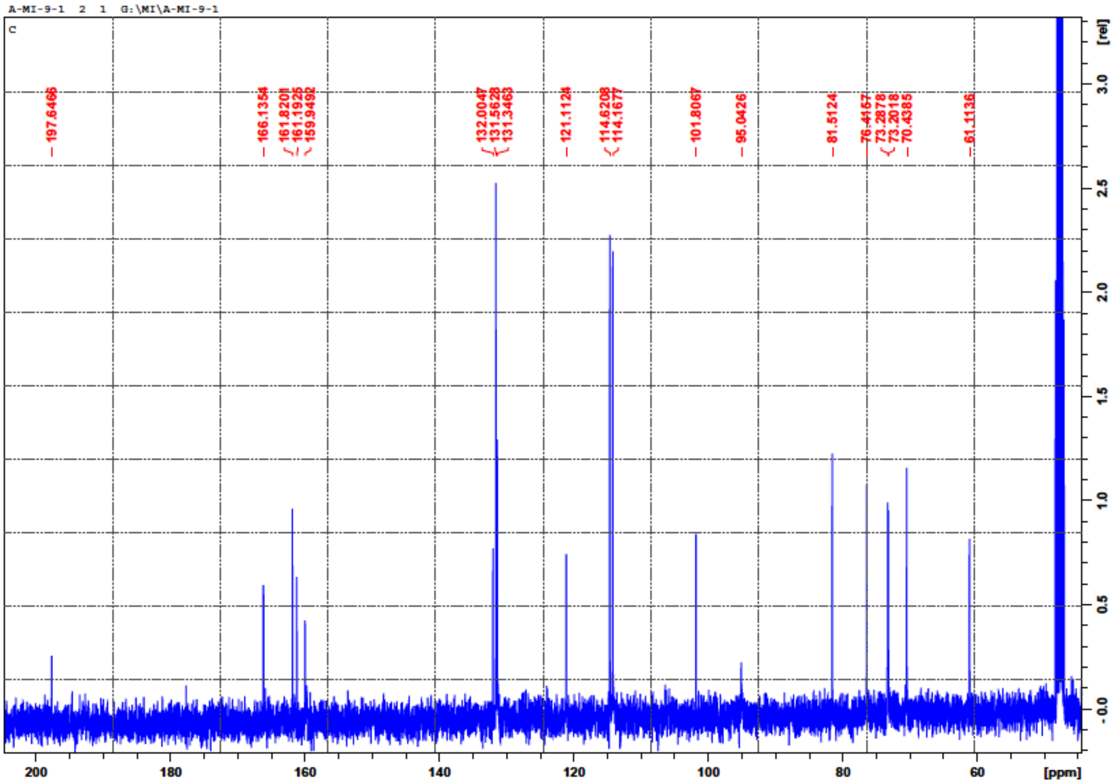

Supplementary figure S 9: ^{13}C-NMR Spectrum of Compound **9-1** (100 MHz, CD3OD)

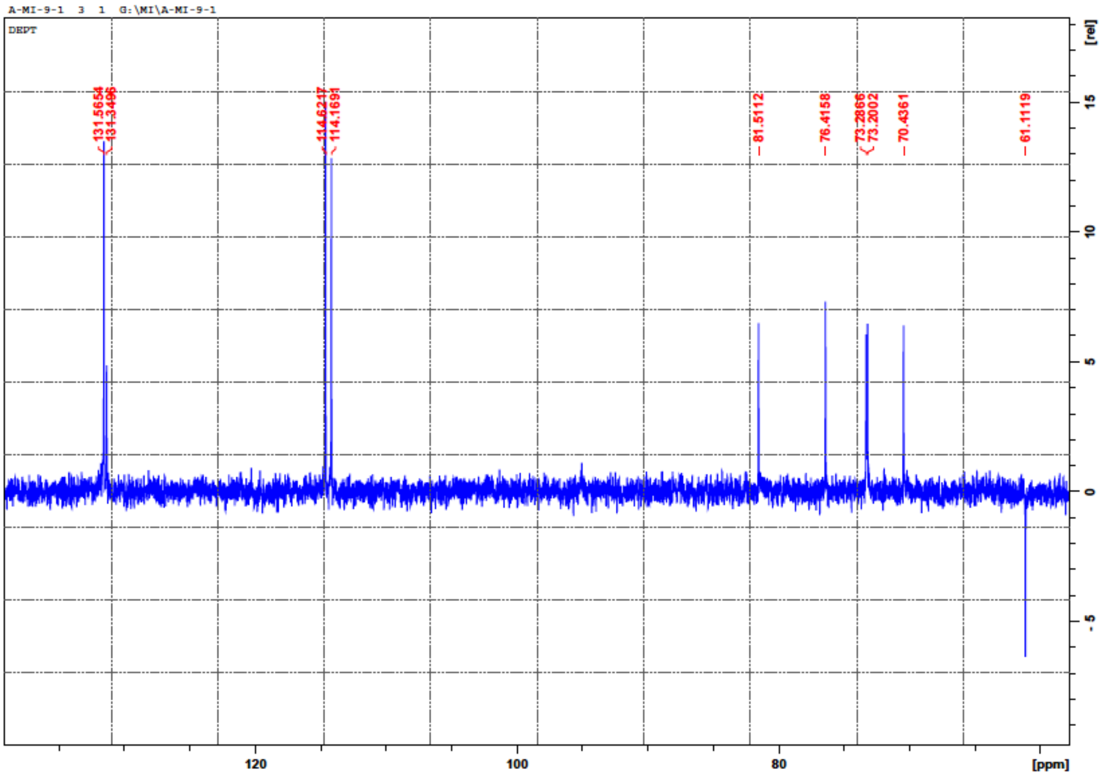

Supplementary figure S10: DEPT Spectrum of Compound **9-1** in CD3OD

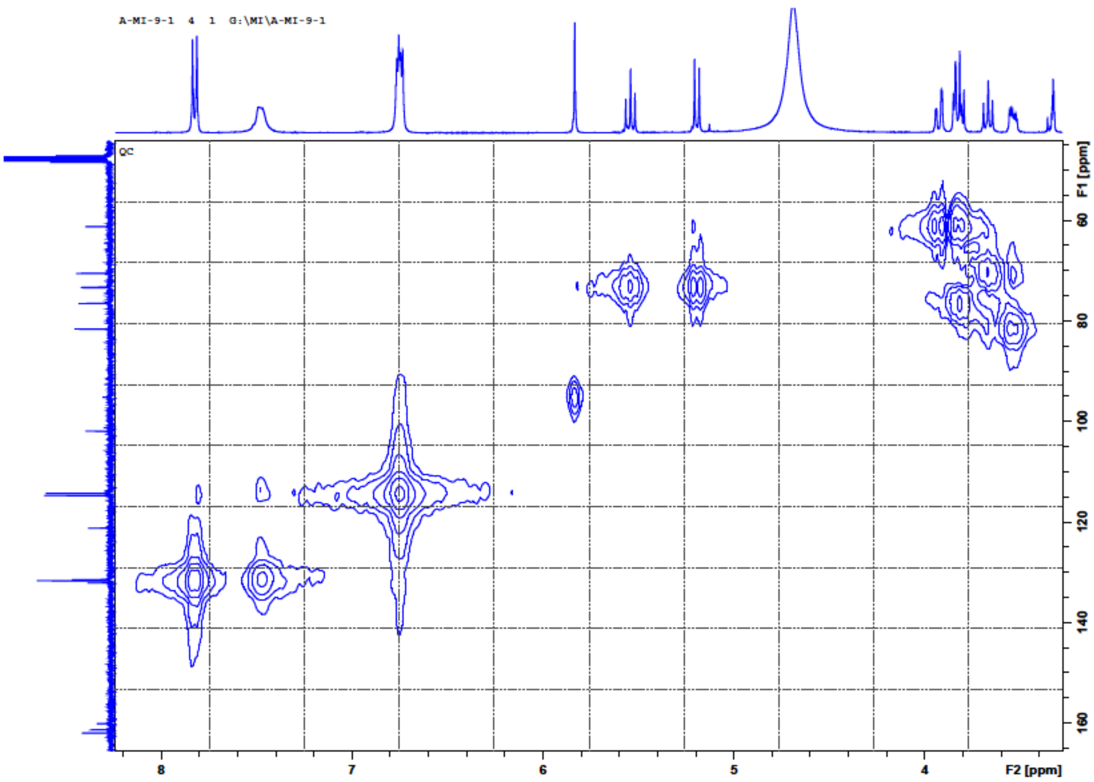

Supplementary figure S11: HMQC Spectrum of Compound **9-1** (400 MHz, CD3OD)

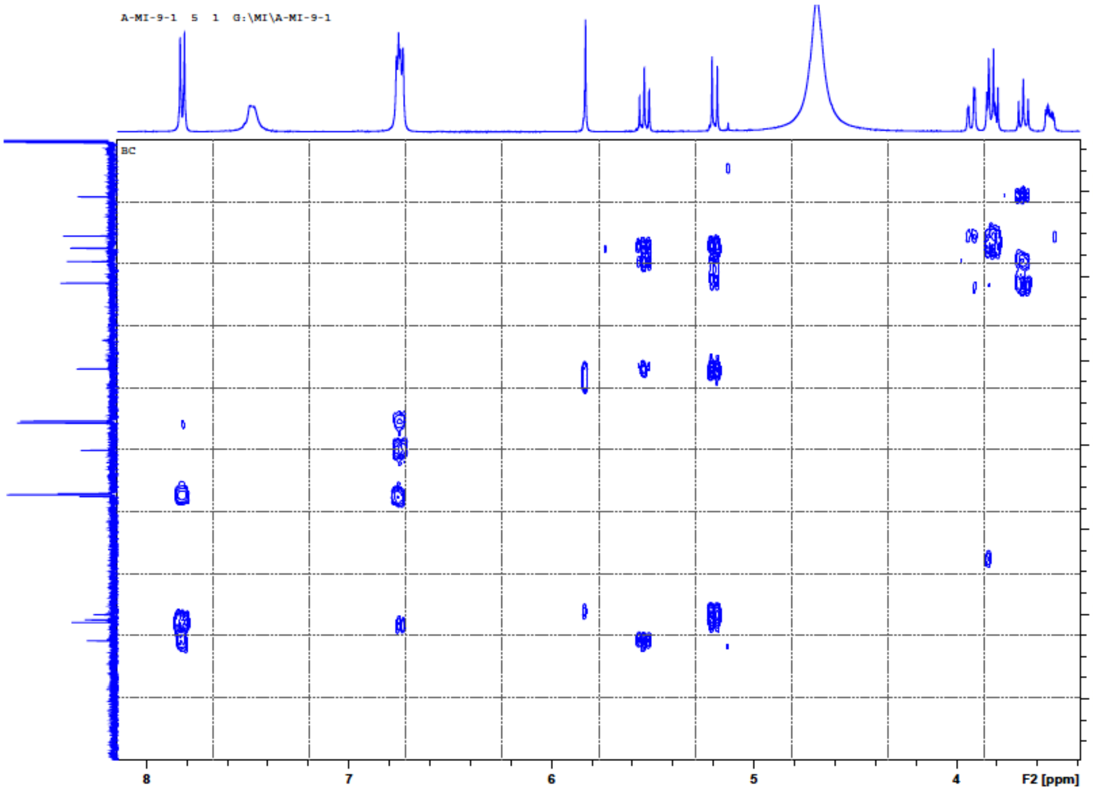

Supplementary figure S12: HMBC Spectrum of Compound **9-1** (400 MHz, CD3OD)

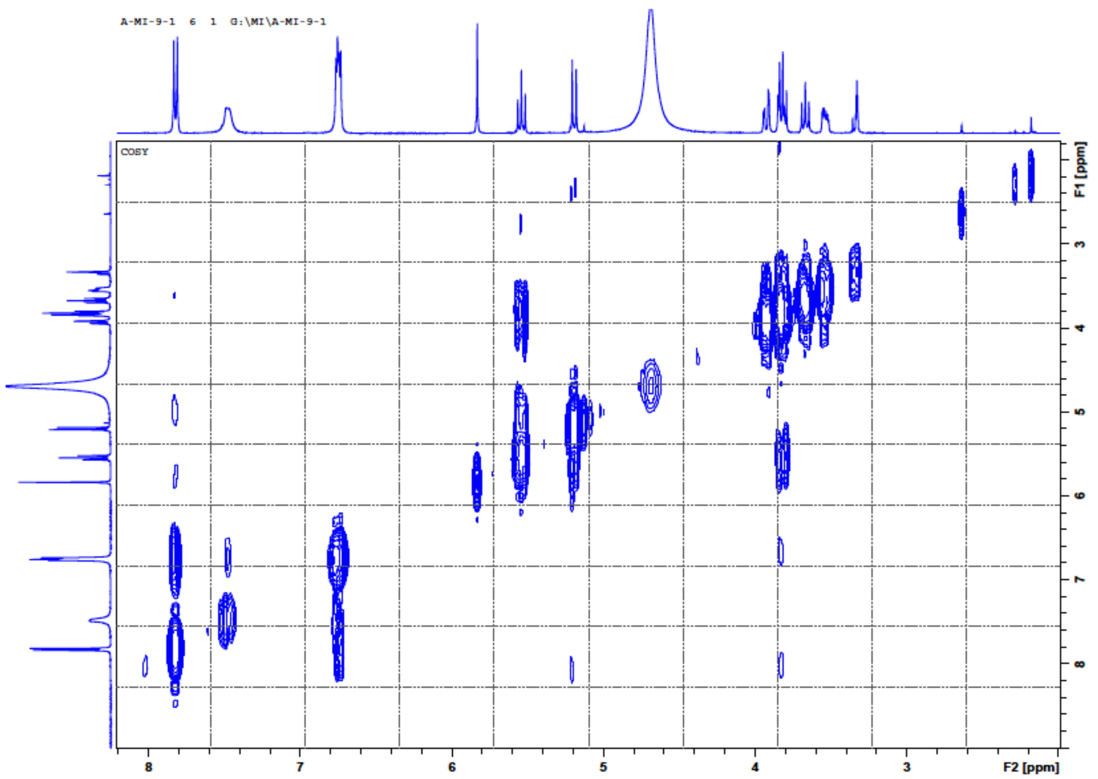

Supplementary figure S13: COSY Spectrum of Compound **9-1** (400 MHz, CD3OD)

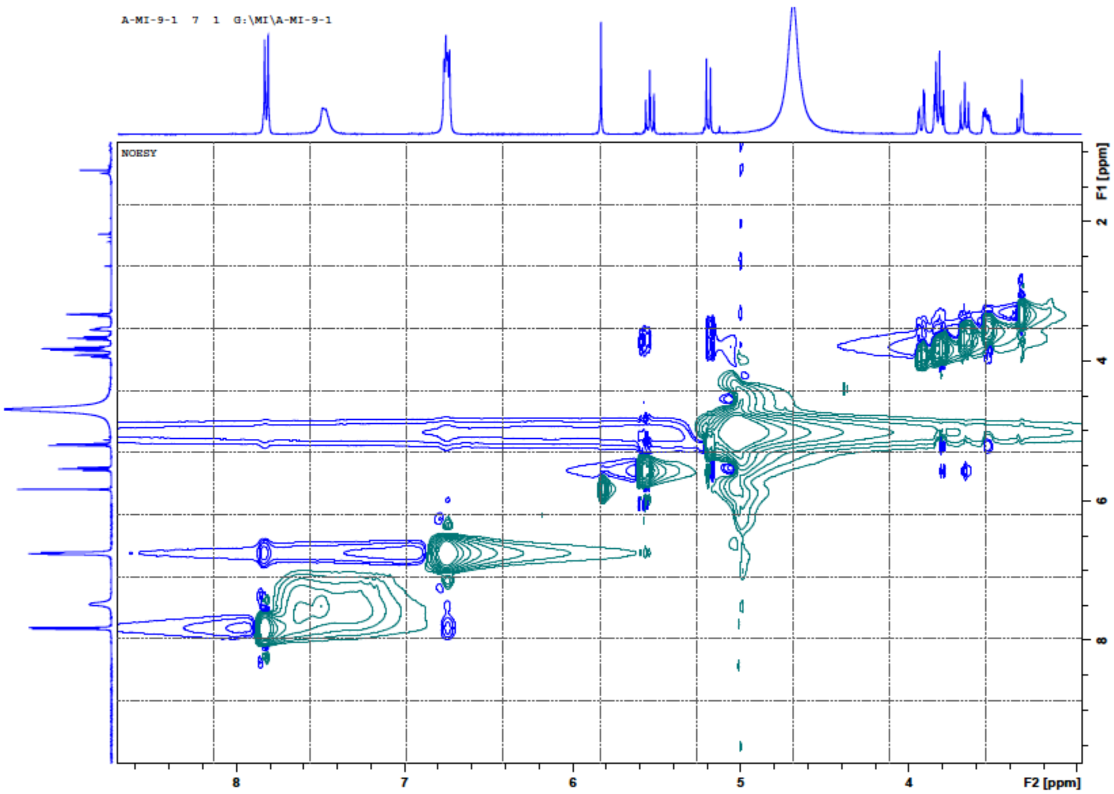

Supplementary figure S14: NOESY Spectrum of Compound **9-1** (400 MHz, CD3OD)

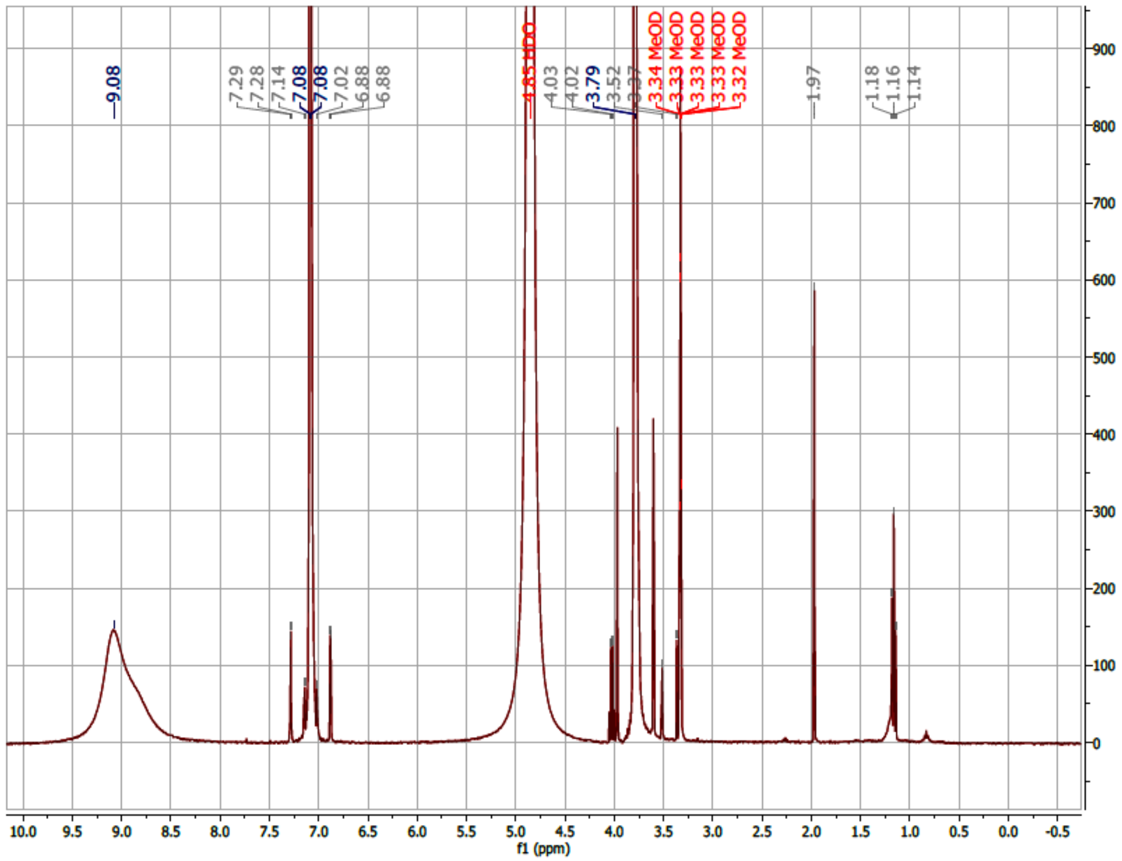

Supplementary figure S15: ^{1}H-NMR Spectrum of Methyl gallate (400 MHz, CD3OD)

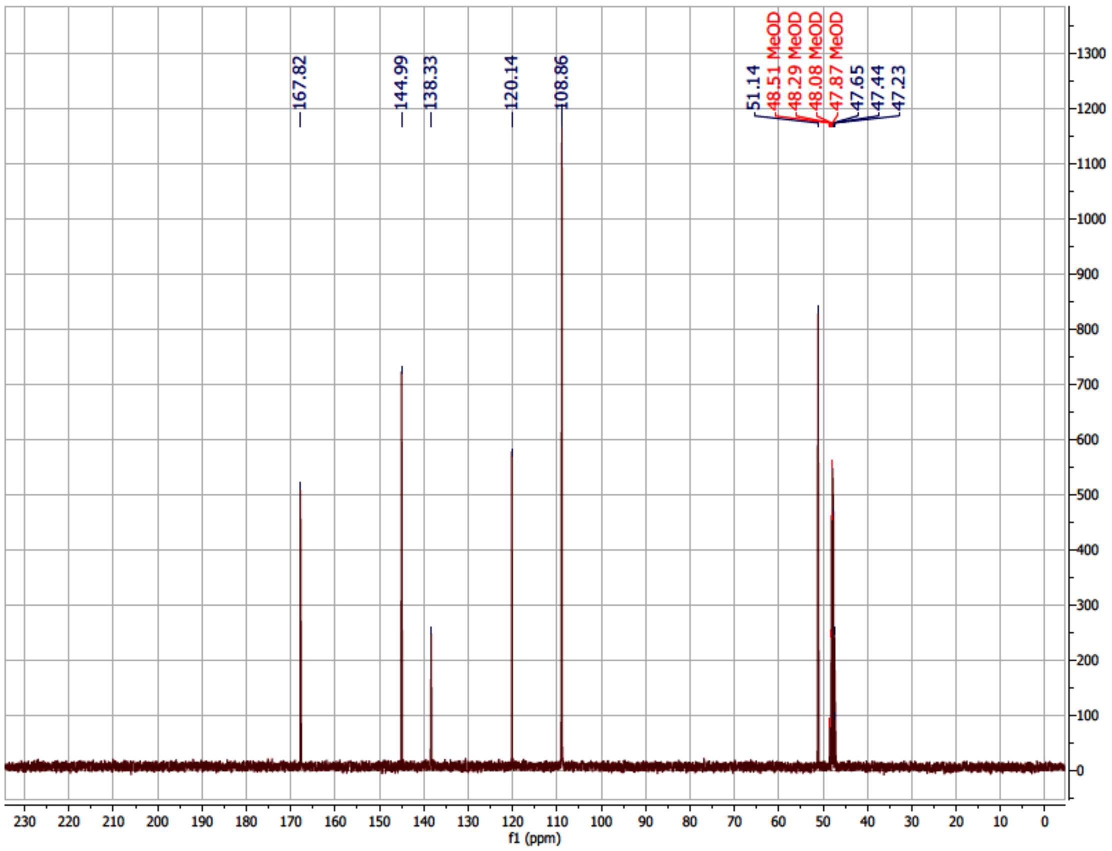

Supplementary figure S16: ^{13}C-NMR Spectrum of Methyl gallate (100 MHz, CD3OD)

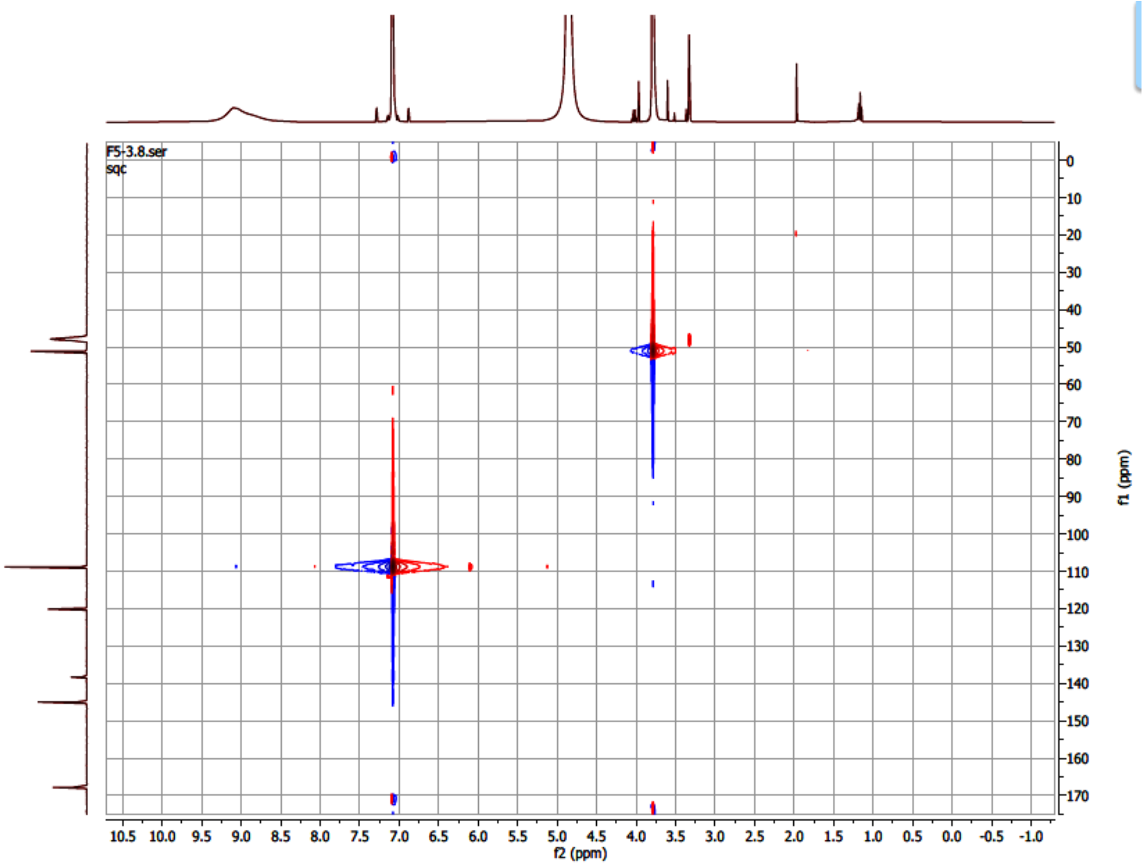

Supplementary figure S17: HMQC Spectrum of Methyl gallate (400 MHz, CD3OD)

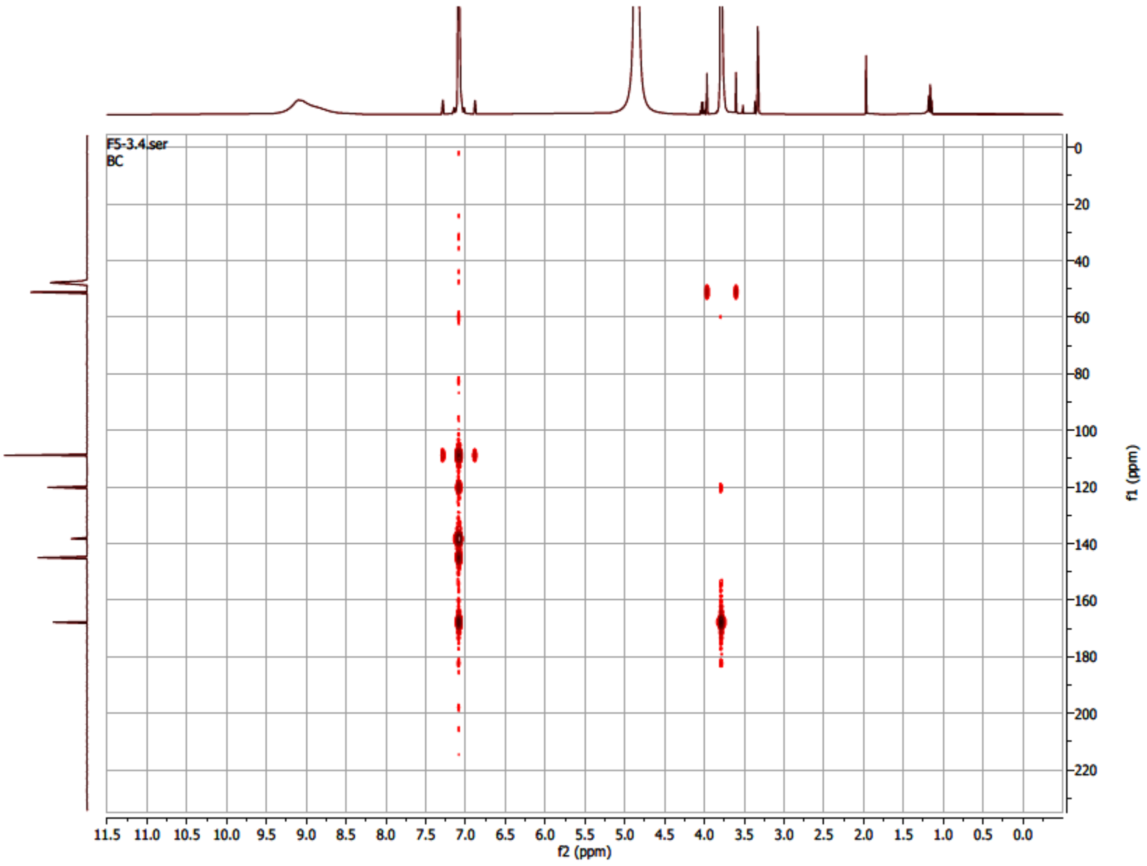

Supplementary figure S18: HMBC Spectrum of Methyl gallate (400 MHz, CD3OD)

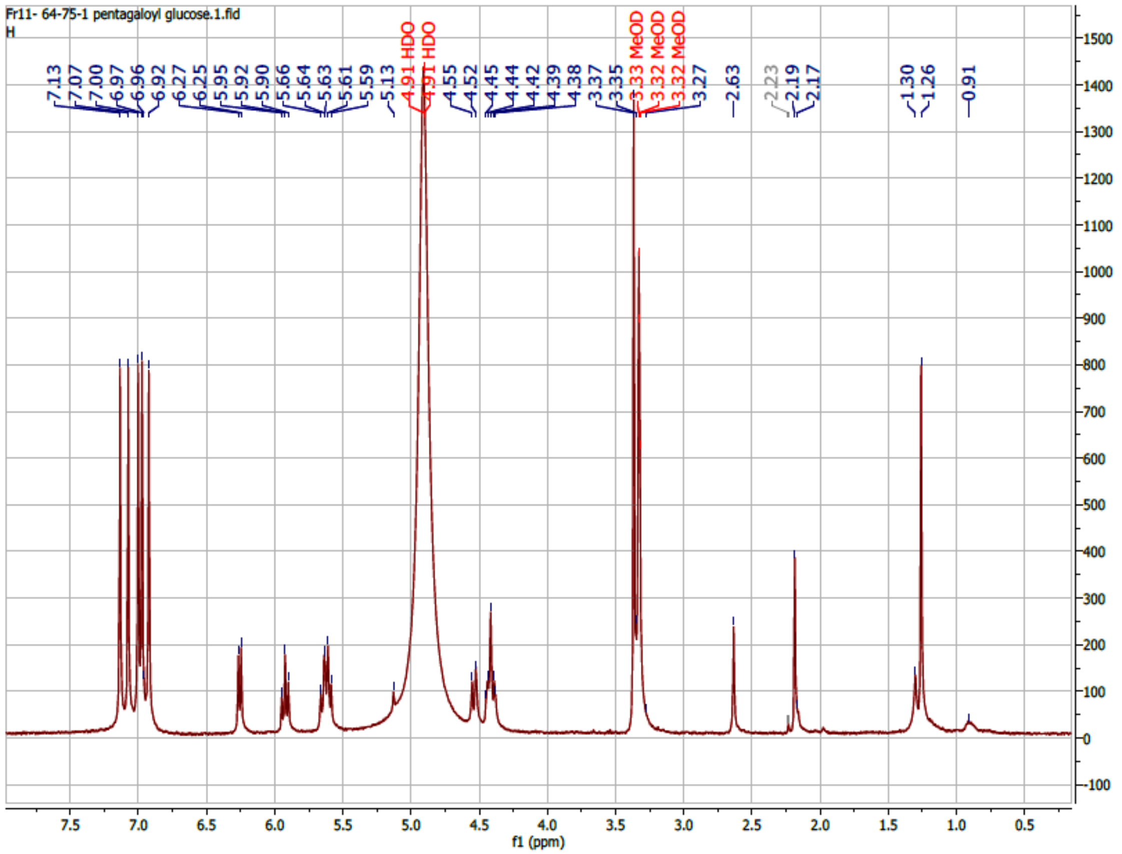

Supplementary figure S19:¹H-NMR Spectrum of pentagalloyl glucoside (400 MHz, CD3OD)

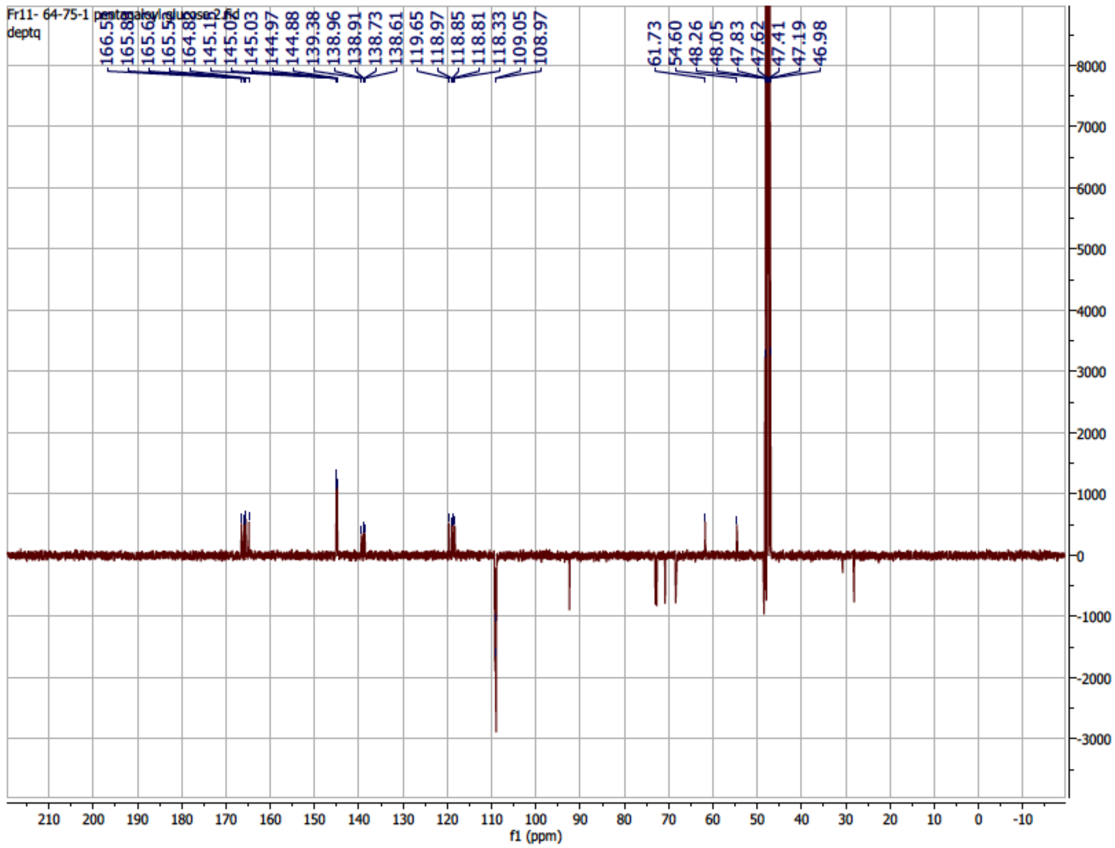

Supplementary figure S20: ^{13}C-NMR Spectrum of pentagalloyl glucoside (100 MHz, CD3OD)

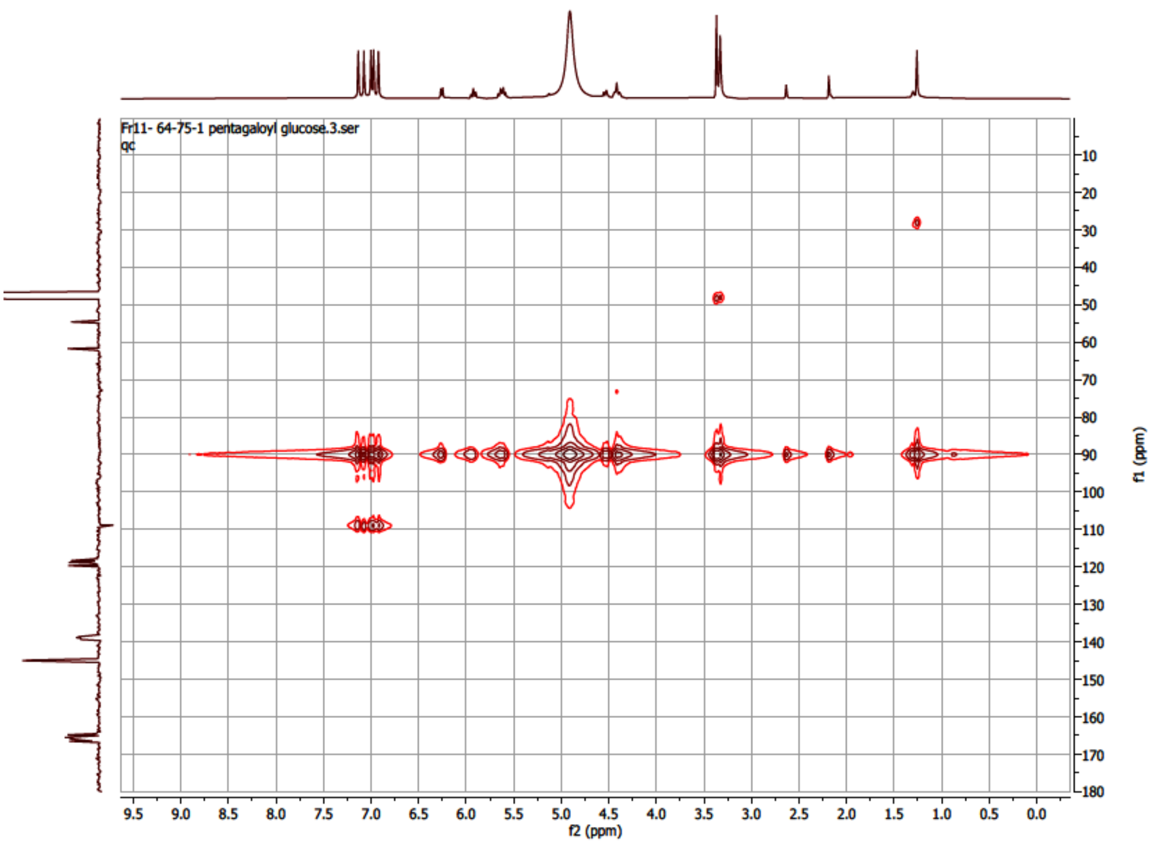

Supplementary figure S21: HMQC Spectrum of pentagalloyl glucoside (400 MHz, CD3OD)

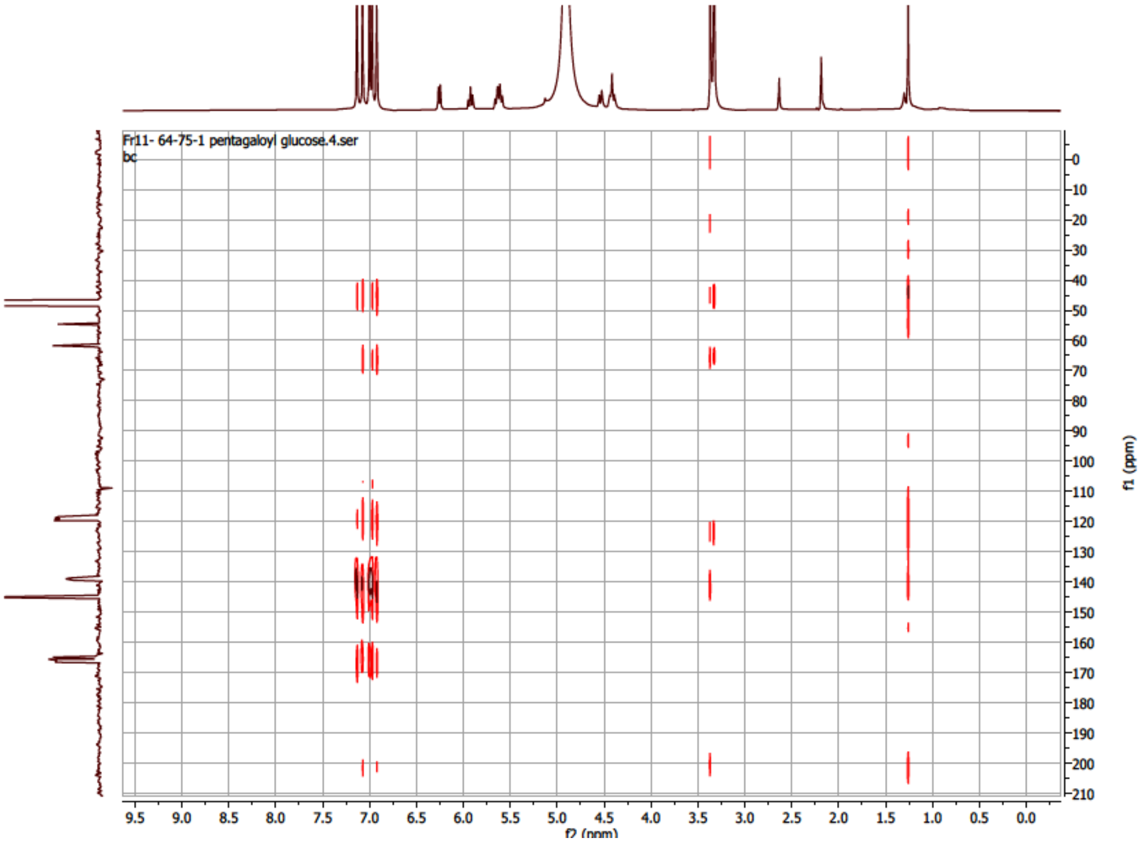

Supplementary figure S22:HMBC Spectrum of pentagalloyl glucoside (400 MHz, CD3OD)

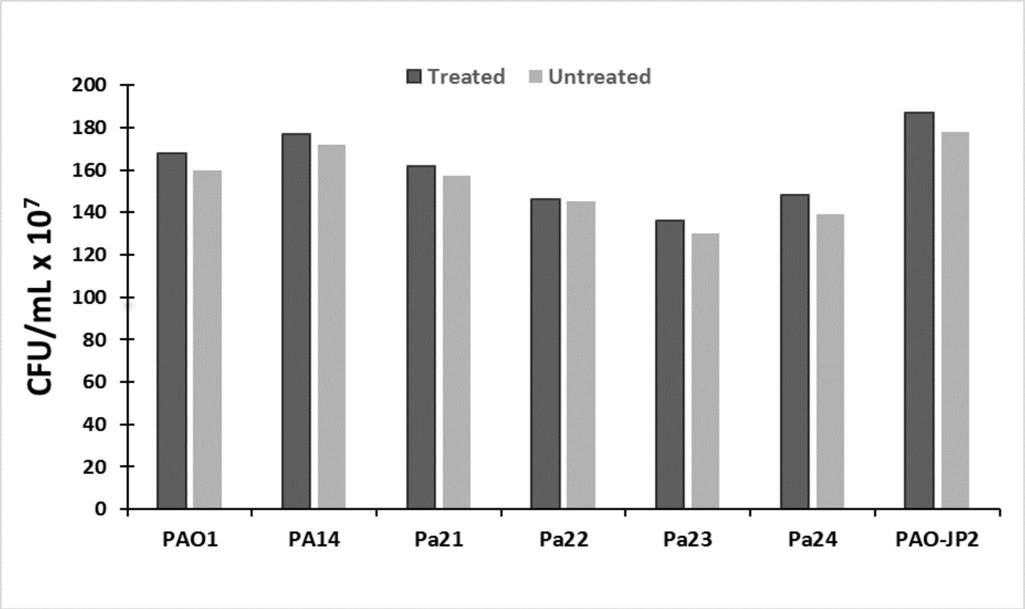

Supplementary figure S23: Bacterial count of *P. aeruginosa* with and without 1/2 MIC of methyl gallate.
